# Molecular epidemiological study of germline *APC* variant associated with hereditary gastrointestinal polyposis in dogs: current frequency in Jack Russell Terriers in Japan and breed distribution

**DOI:** 10.1186/s12917-022-03338-w

**Published:** 2022-06-18

**Authors:** Kyoko Yoshizaki, Akihiro Hirata, Hiroyuki Matsushita, Masahiro Sakaguchi, Wakana Yoneji, Keishi Owaki, Hiroki Sakai

**Affiliations:** 1 Laboratory of Veterinary Pathology, Joint Department of Veterinary Medicine, Faculty of Applied Biological Sciences, Gifu University, 1-1 Yanagido, Gifu, 501-1193 Japan; 2 Present Address: Department of Veterinary Pathology, Joint Faculty of Veterinary Medicine, Yamaguchi University, 1677-1 Yoshida, Yamaguchi, 753-8511 Japan; 3 Laboratory of Microbiology I, Department of Veterinary Medicine, Azabu University, 1-17-71 Fuchinobe, Chuo-ku, Sagamihara-shi, Kanagawa, 252-5201 Japan; 4 Present Address: Institute of Tokyo Environmental Allergy, 1-33-18 Hakusan, Bunkyo-ku, Tokyo, 113-0001 Japan; 5 Nara Animal Referral Clinic, 5-20-7 Mitsugarasu, Nara, 631-0061 Japan

**Keywords:** Hereditary gastrointestinal polyposis, Jack Russell Terrier, Germline *APC* variant, Epidemiology, Variant allele frequency, Breed distribution, Gastric cancer, Colorectal cancer

## Abstract

**Background:**

Cases of gastrointestinal (GI) neoplastic polyps in Jack Russell Terriers (JRTs) have increased in Japan since the late 2000s. We recently demonstrated that JRTs with GI polyps heterozygously harbor an identical germline variant in the *adenomatous polyposis coli* (*APC*) gene, c.[462_463delinsTT]; therefore, this is an autosomal dominant hereditary disease. We conducted a molecular epidemiological study to explore the current frequency of the *APC* variant in JRTs in Japan and the breed distribution of this disease.

**Results:**

Peripheral blood samples from 792 JRTs were collected at 93 veterinary hospitals in Japan in 2020. Using an established polymerase chain reaction-restriction fragment length polymorphism assay, the germline *APC* variant was detected in 15 JRTs, with an overall frequency of 1.89%. The frequency was not significantly different for sex, age, and coat type criteria. Notably, the variant carriers had a current or previous history of GI neoplastic polyps, providing further evidence of the association of the germline *APC* variant with GI polyposis. Pedigree analysis of carrier dogs revealed that the germline *APC* variant was no longer confined to a few specific families but was widely spread among JRTs in Japan. Furthermore, some ancestors of the carriers were from Australia or New Zealand, suggesting the possible presence of carriers in countries other than Japan. Next, we retrospectively investigated the germline *APC* variant status of dogs with GI epithelial tumors using genomic DNA samples extracted from archived pathological specimens (28 purebred dogs of 14 breeds and four mixed-breed dog), as well as those stored in a canine genome bank (38 dogs of 18 breeds and a mixed-breed dogs). In total, 66 purebred dogs of 25 breeds, including another four JRTs, and five mixed-breed dogs were examined. While three variant carriers were found in JRTs, the germline *APC* variant was not detected in any of the other breeds.

**Conclusion:**

The current frequency of the germline *APC* variant was approximately 2% in JRTs in Japan and the frequency remained roughly flat during the last 15 years. In addition, hereditary GI polyposis associated with the variant was virtually specific to JRTs.

**Supplementary Information:**

The online version contains supplementary material available at 10.1186/s12917-022-03338-w.

## Background

In dogs, breed predisposition often provides the first clue to the discovery of a novel hereditary disorder [1]. Although gastrointestinal (GI) epithelial tumors are rare in dogs [2, 3], the number of cases of Jack Russell Terriers (JRTs) with GI neoplastic polyps has recently increased in Japan [4–6] despite a lack of a corresponding increase in JRT population [7]. In a recent study, we demonstrated that JRTs with GI polyps harbor an identical germline variant in the *adenomatous polyposis coli* (*APC*) gene, c.[462_463delinsTT] (GenBank ID: LC598892.1) in a heterozygous state; thus, this is an autosomal dominant hereditary disorder (OMIA ID 001916–9615) [6]. JRTs affected by hereditary GI polyposis develop solitary or multiple neoplastic polyps in the GI tract, and most lesions are histopathologically diagnosed as adenomas or adenocarcinomas [6]. These polyps occur primarily in the stomach and large intestine, with a predilection for the gastric antrum and rectum, and multiple lesions can develop simultaneously at both locations [6]. Early onset and frequent recurrence of GI polyps are also features of hereditary GI polyposis [6]. Based on similarities in the responsible genes and disease phenotypes, this disease can be considered a canine counterpart of familial adenomatous polyposis in humans [8].

When compared with dogs affected with sporadic GI cancers, JRTs with hereditary GI polyposis are expected to have longer survival times, with reported 1- and 2-year survival rates of 100%; but at the same time, they have an increased lifelong risk of recurrence [6]. Therefore, it is critical for veterinary clinicians to distinguish cases of hereditary GI polyposis from those of sporadic GI cancer to provide appropriate treatment and accurately predict prognosis. Once a disease-causing variant is identified, it is possible to develop specific genetic testing for the target variant. In a recent study, we established polymerase chain reaction (PCR)-based genotyping assays capable of detecting the germline *APC* variant [9]. These assays can facilitate not only clinical diagnosis, but also large-scale epidemiological studies of this novel hereditary disease.

Our previous retrospective analysis revealed that cases of JRTs with hereditary GI polyposis began to increase in the late 2000s, and most affected JRTs were born in the first decade of the 2000s [6], suggesting that the germline *APC* variant could have spread among JRTs in Japan during this period. However, the extent to which germline *APC* variant spread in JRTs remains unexplored. A large-scale epidemiological study would determine current frequency and fluctuation of the *APC* variant among JRTs during the last one or two decades.

According to well-established online databases [10–13], there are currently hundreds of hereditary diseases with known causative gene variants in dogs. While many of these diseases occur at a greater frequency in a single breed or several related breeds, some disorders are common to multiple breeds [1, 13]. Recently, Donner et al. investigated 18,000 purebred dogs among 330 different breeds using a DNA panel screening test, testing for 152 known genetic variants, and clearly demonstrated that several known disease-associated variants were more widespread across different breeds than previously recognized [14]. However, this study validated the fact that several disease-associated variants still show complete or virtually complete breed-specificity [14]. Therefore, it is necessary to determine whether this novel hereditary disease is specific to JRTs.

In the present study, using the established genotyping assays [9], we explored the current frequency of the germline *APC* variant associated with hereditary GI polyposis in JRTs in Japan and investigated the breed distribution of this disease by retrospectively examining archived samples of dogs with GI epithelial tumors.

## Results

### Epidemiological survey of JRTs

#### Current frequency of germline *APC* variant associated with hereditary GI polyposis in JRTs

**Fig. 1:**
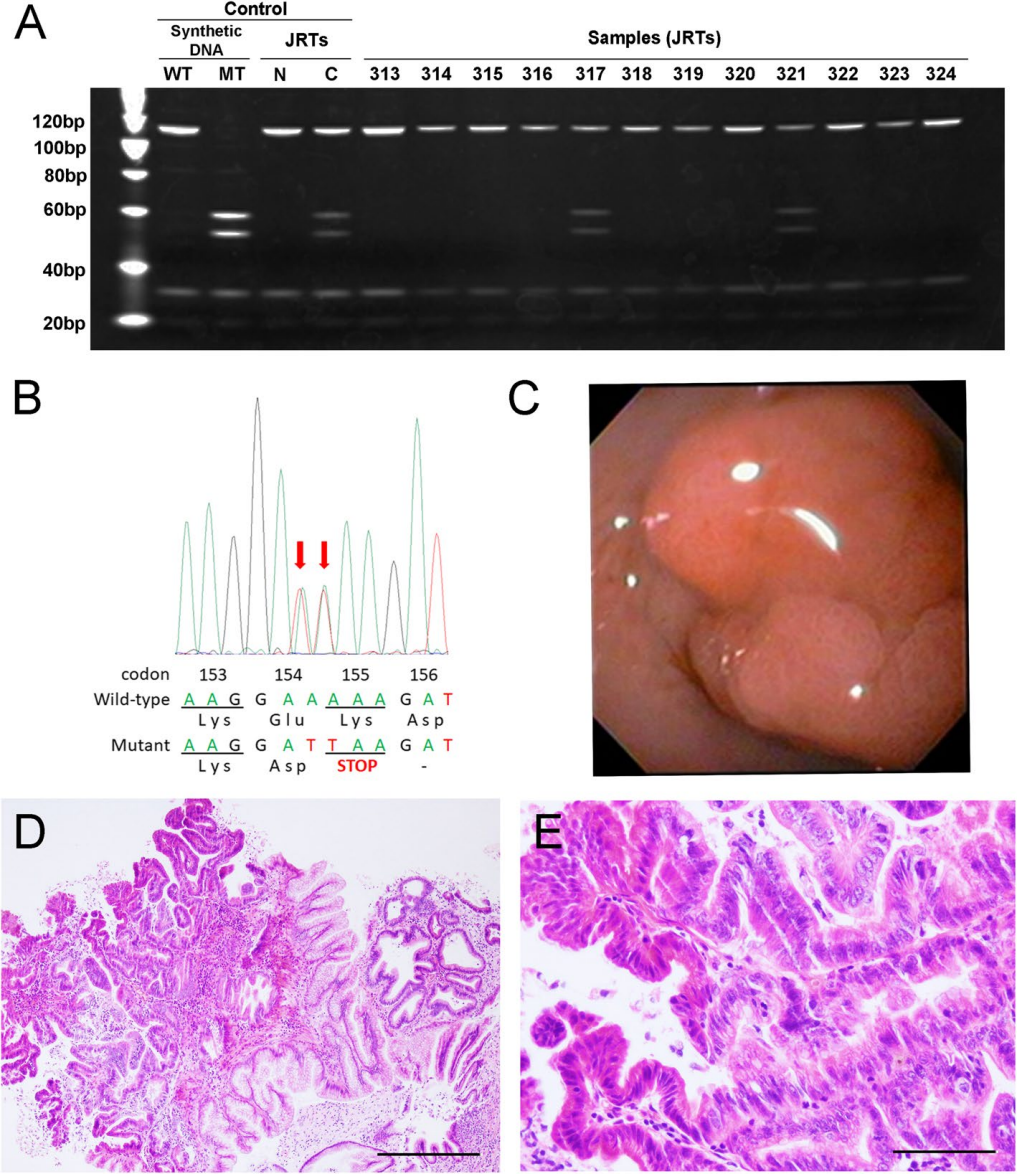
Epidemiological survey of Jack Russell Terriers. **A** PCR-RFLP assay. Representative result of polyacrylamide gel electrophoresis of *Mse*I-digested PCR products. Synthetic wild-type and mutant DNA and DNA samples of previously detected carrier and non-carrier JRTs are used as controls. Case nos. JRT317 and JRT321 are determined to be *APC* variant carriers based on the presence of bands at 51 and 57 bp derived from *APC* variant allele. WT, wild-type; MT, mutant type; N, non-carrier; C, carrier. Full-length gel image is provided in Supplementary Fig. 1A. **B** PCR-direct sequencing. Representative result of a carrier JRT, case no. JRT221. DNA sequence of codons 153–156 in the *APC* gene determined by PCR-direct sequencing. The red arrows indicate 2-bp substitution at codons 154 and 155. See also Supplementary Fig. 2A. **C** Endoscopic appearance of polyps developed in antrum of the stomach in an *APC* variant carrier, JRT152. **D**, **E** Histopathology of biopsied samples from adenocarcinoma in the gastric antrum of JRT152. **E** is higher magnification of (**D**). Bars = 200 µm (**D**), 50 µm (**E**)

The peripheral blood of 792 JRTs collected at 93 veterinary clinics in Japan in 2020 was analyzed. An established PCR-restriction fragment length polymorphism (PCR-RFLP) assay successfully determined the *APC* genotypes of all examined JRTs, and 15 JRTs were found to be carriers of the germline *APC* variant (Fig. 1A). The presence of the *APC* variant, c.[462_463delinsTT], was validated by PCR-direct sequencing for all 15 JRTs (Fig. 1B). The overall frequency of germline *APC* variant in JRTs was 1.89%. There was no significant difference in frequency depending on sex and coat type (Table 1). When divided into 5-year age groups, the frequency of the *APC* variant was around 2% in all age groups without significant fluctuations.

**Table 1:** Frequency of germline *APC* variant in Jack Russell Terriers

	Total number of examined dogs	Number of *APC* variant carriers (%)	*P* value
***Total***	792	15 (1.89%)	
***Birth year***			0.72671914
2001–2005	57	0 (0%)	
2006–2010	272	5 (1.84%)	
2011–2015	286	6 (2.10%)	
2016–2020	175	4 (2.29%)	
Unknown	2		
***Sex***			0.771578924
Male	399	7 (1.75%)	
Female	393	8 (2.04%)	
***Coat type***			0.704531295
Smooth	396	9 (2.27%)	
Broken	212	3 (1.42%)	
Rough	129	3 (2.33%)	
Unkown	55		

#### Medical history and current symptoms of *APC* variant carriers

Interviews with veterinarians and dog owners revealed information on the current and previous medical histories and clinical symptoms of the detected *APC* variant carriers. As expected, of ten carriers with available information, five developed GI cancers before or after the present epidemiological survey (Table 2). Case no. JRT216 had a previous history of repeated occurrence of rectal adenocarcinoma at the age of 4 and 6 years. Despite the lack of histopathological examination, the dog had recurrence of rectal polyp at age 7. As for case no. JRT152, the 8-year-old female dog was presented to a veterinary clinic with blood-tingled vomitus during the present epidemiological study, and polyps were endoscopically detected in the cardia and antrum of the stomach (Fig. 1C). Based on histopathological examination of the biopsy specimen, both gastric polyps were diagnosed as adenocarcinomas (Fig. 1D, E). In case no. JRT203, the detection of the germline *APC* variant in the present study led to the early detection of gastric cancer. The dog was prone to vomiting, and therefore in response to the positive genetic test result, she underwent endoscopic examination at the age of 6 years. An antrum polyp was detected and histopathologically diagnosed as an adenocarcinoma. Furthermore, two dogs developed GI cancers after the survey. The first case was of JRT317; the dog had no GI symptoms at the time of blood sampling. Six months later, bloody stool was observed, and rectal adenocarcinoma was surgically resected when the dog was 3 years of age. The second dog, case no. JRT342, had a history of intermittent vomiting for many years; when the dog was 11 years of age, endoscopic biopsy revealed adenocarcinoma in the antrum of the stomach. Three months later, abdominal surgery was performed, but tumor resection was abandoned due to adhesion to adjacent organs, including the pancreas. During abdominal surgery, the dog underwent Billroth II gastrojejunostomy to relieve the gastric outlet obstruction but died 2 months later.

**Table 2:** Profile and clinical information of Jack Russell Terriers with germline *APC* variant detected in prevalence survey

No	Case No	Birth month	Sex	Coat type	Age	Clinical History (method of excision)	Clinical symptoms
1	JRT055	2010/04	Male	Smooth	10 y 7 m		vomiting, anorexia
					10 y 8 m	Deceased	vomiting, anorexia^a^
2	JRT123	2009/06	Female (spayed)	Smooth		N.D.	N.D.
3	JRT152	2012/01	Female	Smooth	8 y 3 m	Antrum and cardia of the stomach: adenocarcinomas (endoscopic biopsy)	vomiting, hematemesis
4	JRT196	2017/05	Male	Smooth	2 y 10 m	( −)	bloody stools, soft stools, vomiting, hematemesis
5	JRT203	2014/10	Female (spayed)	Broken	Earlier age		bloody stools, melena, diarrhea
					6 y 2 m	Antrum of the stomach: adenocarcinoma (endoscopic biopsy)	vomiting
6	JRT216	2013/03	Male (castrated)	Smooth	4 y 10 m	Rectum: adenocarcinoma (pull-through)	bloody stools
					6 y 1 m	Rectum: adenocarcinoma (endoscopic biopsy)	bloody stools, frequent stools
					7 y 0 m	Rectum: polyp^b^	
7	JRT221	2016/12	Female (spayed)	Smooth		( −)	( −)
8	JRT264	2014/05	Male (castrated)	Rough		N.D.	N.D.
9	JRT317	2017/01	Female (spayed)	Smooth	3 y 8 m		bloody stools
					3 y 10 m	Rectum: adenocarcinoma (surgery)	
10	JRT321	2009/09	Female (spayed)	Broken	10 y 8 m	( −)	diarrhea, melena
11	JRT342	2009/03	Female (spayed)	Rough	Earlier		vomiting
					11 y 1 m	Antrum of the stomach:adenocarcinoma (endoscopic biopsy)	vomiting
					11 y 4 m	Antrum of the stomach:adenocarcinoma	
					11 y 6 m	Deceased	
12	JRT447	2017/03	Male (castrated)	Rough	3 y 3 m	( −)	soft stools
13	JRT535	2015/07	Male (castrated)	Smooth		N.D.	N.D.
14	JRT671	2011/10	Male (castrated)	Smooth		N.D.	N.D.
15	JRT697	2007/03	Female (spayed)	Broken		N.D.	N.D.

*N.D.* No data, *y* Years, *m* Months

^a^The dog showed serious vomiting and anorexia just before death

^b^The polyp was not examined histopathologically

Our previous study showed that vomiting and bloody stool are the most frequent symptoms in JRTs after the onset of hereditary GI polyposis [6]. Despite the absence of a clinical examination for GI cancers, GI symptoms were present in four carriers (Table 2), of which JRT056 showed serious GI symptoms before death from an uncertain cause at the age of 10 years. Among the detected variant carriers with the available information, only JRT221 remained asymptomatic until the age of 3.5 years.

#### Pedigree analysis of the *APC* variant carriers

Pedigree certificates of nine carriers were available with the cooperation of the dog owners, and they contained the information of three generations in the ancestry of each dog. The blood relationships of the carriers were analyzed along with five additional previous cases (Supplemental Table 1) [6].

**Fig. 2:**
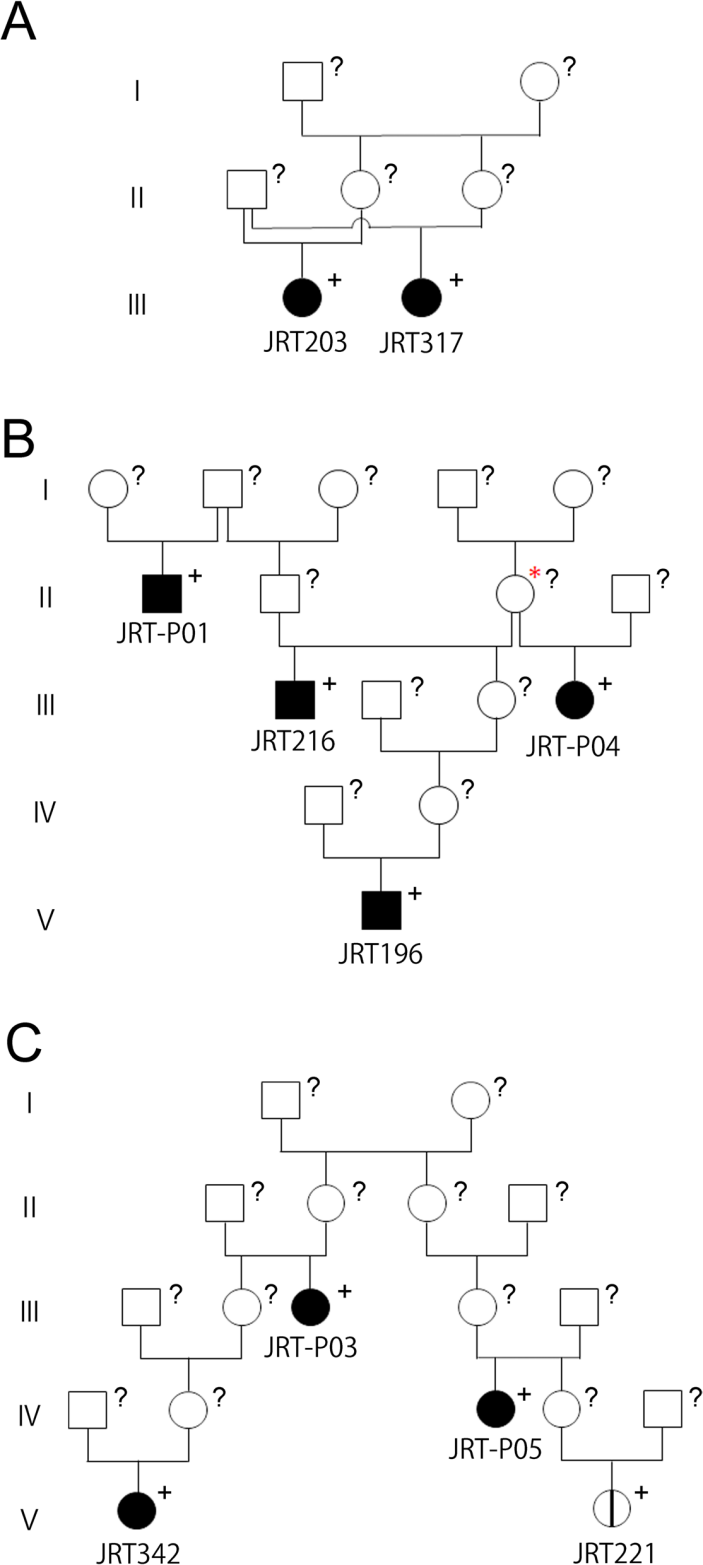
Blood relationships of Jack Russell Terriers with germline *APC* variant. Family tree constructed according to pedigree certificates of *APC* variant carriers. **A** Family tree containing paternal half-sisters, JRT203 and JRT317. **B** Family tree containing four carriers, JRT196, JRT216, JRT-P01, and JRT-P04. Asterisk indicates common ancestor of JRT196, JRT216, and JRTP04. **C** Family tree containing four carriers, JRT221, JRT342, JRT-P03, and JRT-P05. These carrier dogs are descended from a common breeding pair described in generation I. Squares and circles show males and females, respectively. The black pattern and vertical line indicate dogs with and without history of GI neoplastic polyps, respectively. The white pattern indicates dogs with unknown medical history. + , *APC* variant carrier; ?, *APC* genotype unknown

Three groups with blood relationships were identified among the carriers. The first group consisted of paternal half-sisters, JRT203 and JRT317, and their mothers were also non-littermate full-sisters (Fig. 2A). In the second group, JRT216 was a maternal half-brother of JRT-P04, and JRT196 was also a descendant of their common mother dog (Fig. 2B). The last group contained four JRTs that descended from a common breeding pair (Fig. 2C). It should also be noted that the remaining four carriers, JRT152, 447, 671 and JRT-P02, had no relationship with any other carriers in the past three generations, indicating that the germline *APC* variant was no longer confined to a few specific families and was widely spread among JRTs in Japan.

The pedigree analysis also revealed that many JRTs from Australia and New Zealand were present among the ancestors of the *APC* variant carriers, mainly in the great-grandparents’ generation (Table 3).

**Table 3:** Ancestor dogs born in countries other than Japan

No	Case No	Numbers of ancestral dogs born in countries other than Japan in each generations (n)		
		Parents	Grandparents	Great-grandparents
1	JRT152	0	0	2^a^ (Australia *n* = 2)
2	JRT196	0	0	1 (Australia *n* = 1)
3	JRT203	0	0	1 (Australia *n* = 1)
4	JRT216	0	0	1 (Australia *n* = 1)
5	JRT221	0	0	2 (Australia *n* = 2)
6	JRT317	0	0	2 (Australia *n* = 2)
7	JRT342	0	0	2 (Australia *n* = 2)
8	JRT447	0	0	0
9	JRT671	0	0	2 (Australia *n* = 2)
10	JRT-P01	0	1 (Australia *n* = 1)	4 (Australia *n* = 3, Not specified^b^ *n* = 1)
11	JRT-P02	0	2 (Australia *n* = 1, New Zealand *n* = 1)	7 (Australia *n* = 1, New Zealand *n* = 2, Not specified^b^ *n* = 4)
12	JRT-P03	1 (Australia *n* = 1)	2 (Australia *n* = 2)	7 (Australia *n* = 5, Not specified^b^ *n* = 2)
13	JRT-P04	0	0	2 (Australia *n* = 2)
14	JRT-P05	0	1 (Australia *n* = 1)	4 (Australia *n* = 3, Not specified^b^ *n* = 1)

^a^The two dogs were common parents of two dogs in the grandparents’ generation: brother-sister mating were conducted in the grandparents’ generation

^b^The dogs were parents of dogs born in Australia or New Zealand despite the lack of specification of their national origin in pedigree certificates in the grandparents' generation

### Breed distribution of hereditary GI polyposis in dogs

To investigate breed distribution of hereditary GI polyposis, germline *APC* variant status of dogs with GI epithelial tumors was retrospectively examined using samples obtained from the pathology archive and canine genome bank.

#### Analysis of archived pathological samples

Using formalin-fixed paraffin-embedded (FFPE) specimens, 32 cases were examined, including 28 purebred dogs of 14 different breeds and four mixed-breed dogs (Table 4). JRTs were excluded because they had already been evaluated in our previous study [6]. Characteristics of the examined cases and information on the tumors are summarized in Supplemental Table 1. Except for a single case of gastric tumor, all tumors were located in the small and large intestines (*n* = 13 and 18, respectively). While all tumors in the stomach and small intestine were adenocarcinomas, tumors in the large intestine were diagnosed as adenomas (*n* = 13) or adenocarcinomas (*n* = 5).

**Table 4:** Breeds and numbers of dogs with gastrointestinal tumors examined for germline *APC* variant

Dog breed	n
*FFPE samples (n* = *32)*	
Toy Poodle	5
Chihuahua	4
Miniature Dachshund	4
French Bulldog	3
Labrador Retriever	2
Shih Tzu	2
Bichon Frise	1
Border Collie	1
Bull Terrier	1
Maltese	1
Miniature Schnauzer	1
Welsh Corgi	1
West Highland white terrier	1
Wire Fox Terrier	1
Mixed-breed	4
*Genome bank samples (n* = *39)*	
Miniature Dachshund	12
Jack Russell Terrier	4
Toy Poodle	4
Shih Tzu	3
Chihuahua	2
American Cocker Spaniel	1
Belgian Shepherd Tarvuren	1
Boston Terrier	1
Brittany Spaniel	1
Chinese Crested Dog	1
French Bulldog	1
Golden Retriever	1
Lakeland Terrier	1
Miniature Schnauzer	1
Papillon	1
Shetland Sheepdog	1
Shiba Inu	1
West Highland white terrier	1
Mixed-breed	1

**Fig. 3:**
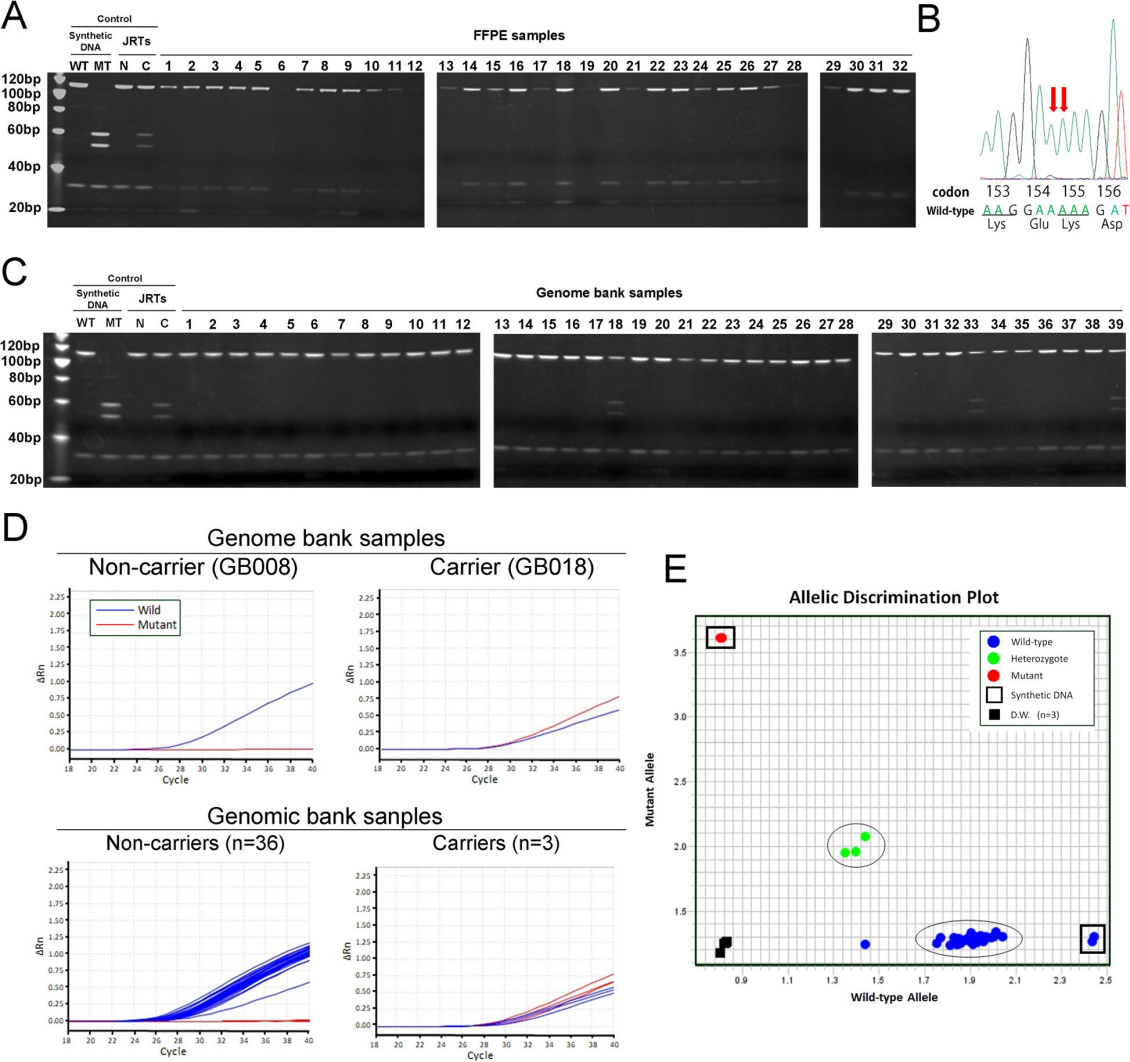
Analysis of breed distribution of germline *APC* variant. Results of genetic testing for germline *APC* variant of dogs with gastrointestinal (GI) epithelial tumors of multiple breeds. **A** PCR-RFLP analysis of FFPE samples. Polyacrylamide gel electrophoresis of *Mse*I-digested PCR products. In the analysis, it is impossible to recognize the clear fragment pattern in some lanes. Synthetic wild-type and mutant DNA and DNA samples of previously detected carrier and non-carrier JRTs are used as controls. Three images cropped from three different gels are grouped and the full-length gel images are provided in Supplementary Fig. 1B. WT, wild-type; MT, mutant type; N, non-carrier; C, carrier. **B** PCR-direct sequencing. Representative result of a FFPE sample, case no. FFPE032. DNA sequences of codons 153–156 in the canine *APC* gene determined by PCR-direct sequencing. The *APC* variant at codons 154 and 155 is absent. See also Supplementary Fig. 2B. **C** PCR-RFLP analysis of genome bank samples. Polyacrylamide gel electrophoresis of *Mse*I-digested PCR products amplified from genome bank samples. In the analysis, three cases, case nos. GB018, GB033 and GB039, are determined to be *APC* variant carriers based on the presence of bands at 51 and 57 bp derived from *APC* variant allele. Synthetic wild-type and mutant DNA and DNA samples of previously detected carrier and non-carrier JRTs are used as controls. Three images cropped from three different gels are grouped and the full-length gel images are provided in Supplementary Fig. 1C. WT, wild-type; MT, mutant type; N, non-carrier; C, carrier. **D** TaqMan duplex real-time PCR. Real-time amplification plots of wild-type (blue curve) and mutant (red curve) *APC* gene copies. Amplification was plotted as VIC and FAM fluorescence intensities (ΔRn value) against cycle numbers. Upper panels show representative results of individual cases of the detected carrier or non-carrier, GB008 and GB018, respectively. Lower panels summarize the results of all examined cases (*n* = 36 and 3 for carrier and non-carrier, respectively). **E** TaqMan duplex real-time PCR. Allelic discrimination plot based on the signal intensity ratio of FAM and VIC at the end points of PCR amplification. Synthetic wild-type and mutant DNAs are located at bottom right and top left corners, respectively, and distilled water used as negative control is at the bottom left corner. Except for one case, non-carrier dogs are clustered together at the bottom right portion. Carrier dogs are located near the diagonal line forming a distinct cluster

*APC* variant status was first analyzed using the PCR-RFLP assay, but clear fragment patterns were not observed for some samples, possibly due to the lower quality of the extracted DNA (Fig. 3A). Subsequently, all samples were also analyzed by PCR-direct sequencing, and the *APC* variant was not detected in any case (Fig. 3B).

#### Analysis of canine genome bank samples

Additional DNA samples were obtained from the canine genome bank at Azabu University, which contained 38 purebred dogs of 18 different breeds including four JRTs and a mixed-breed dog (Table 4). Characteristics of the individual dogs and information on GI cancers are provided in Supplemental Table 1. The cases were affected by adenocarcinomas of the stomach (*n* = 10) or adenomas and adenocarcinomas of the intestine (*n* = 6 and 23, respectively). The registered histopathological diagnoses of tumors in JRTs were gastric adenocarcinoma (*n* = 2) and intestinal adenocarcinoma (*n* = 2).

The PCR-RFLP assays revealed that while three JRTs were carriers of the germline *APC* variant, the remaining 36 dogs were non-carriers (Fig. 3C). A JRT with rectal adenocarcinoma did not harbor the germline *APC* variant and was determined to be a sporadic case. The presence or absence of *APC* variant in all samples was validated using PCR-direct sequencing (data not shown).

Furthermore, the genome bank samples were analyzed by TaqMan duplex real-time PCR assay to validate the practical usefulness of the previously established assay [9]. By taking advantage of high-throughput genotyping, all genome bank samples were analyzed at once. Real-time amplification plots of 6-carboxyfluorescein (FAM) and 2-chloro-7’phenyl-1,4-dichloro-6-carboxy-fluorescein (VIC) fluorescence intensities indicated the genotypes of each case (Fig. 3D), which was consistent with the results of PCR-RFLP and PCR-direct sequencing analyses. In addition, when allelic discrimination analysis was conducted based on the VIC or FAM signal intensity ratio at the endpoints of PCR amplification, the examined samples were divided into two clusters, as expected (Fig. 3E). Although one anomalous sample was excluded from the wild-type cluster, its genotype was readily determined by real-time amplification plot, as with other samples (data not shown).

## Discussion

In the present study, we successfully obtained a sufficient number of blood samples from JRTs without additional invasive procedures by collecting them during the annual test for filarial infection. The overall frequency of the germline *APC* variant was determined to be 1.89% during the screening of 792 JRTs in Japan. As in the previous study with 21 carrier dogs, all of the *APC* variant carriers in the present study were heterozygotes; this is consistent with earlier findings that mice homozygous for *APC* variant allele died during embryonic development [15, 16]. Significantly, the frequency of the germline *APC* variant in JRTs did not substantially change depending on birth year, suggesting that there was no substantial fluctuation in the variant allele frequency during the last 15 years. Once disease-associated gene variants are identified in dogs, it is possible to substantially reduce the prevalence by preventing transmission of the deleterious variant to future generations. It has been reported that several disease variants have been significantly reduced in frequency or eradicated in the general dog population [1, 14]. To control canine hereditary diseases, the difference in the mode of inheritance must be considered; while most canine hereditary diseases have recessive inheritance [1, 13, 17], this is a dominant disease. Therefore, using *APC* variant carriers for breeding is prohibited. However, as hereditary GI polyposis is an adult-onset disease, there is a risk of the germline *APC* variant carrier being inadvertently employed for breeding before disease onset. Therefore, this study suggests compulsory genetic testing for the *APC* variant before breeding to allow quick eradication of this disease.

The present study provides further evidence of the association between germline *APC* variant and GI polyposis in JRTs. As expected, among the *APC* variant carriers with available medical history, half of the JRTs had current and/or previous history of adenocarcinomas of the stomach or large intestine. In addition, consistent with our previous study [6], early onset and recurrent cases were present in the carrier dogs. Notably, the detection of the germline *APC* variant in the present epidemiological study led to the early detection of gastric cancer in a JRT. Considering that genetic testing is less invasive, it would be a better diagnostic option for JRTs with refractory GI symptoms in a clinical setting. Positive results would prompt further examination, such as GI endoscopy. Moreover, in the present study, a follow-up survey of the variant carriers revealed that genetic testing preceded the onset of GI cancer in two cases. This result demonstrates the usefulness of genetic testing for predicting the lifelong risk of GI cancer in JRTs. Considering that initial GI lesions can arise at variable ages, reportedly between 2 and above 10 years, in JRTs with the germline *APC* variant [6], future risk assessment is important for the early detection of onset. In contrast, there were also *APC* variant carriers without a history of GI cancer, although some dogs showed GI symptoms suggestive of cancer development [6]. However, some carriers, including an asymptomatic dog, did not reach the reported average age of GI polyposis onset of 7.7 years [6]. Further follow-up surveys of carriers are required to determine whether the *APC* variant exhibits complete penetrance in JRTs.

Even within the same dog breed, the prevalence of certain hereditary diseases can differ among geographically separated subpopulations [18–20]. While JRT is a popular dog breed in other countries as well, there has been no report of JRTs with hereditary GI polyposis in any country other than Japan. In contrast, pedigree analysis revealed that many JRTs were introduced from Australia and New Zealand in the past three generations of the detected *APC* variant carriers. Taken together, the germline *APC* variant has become regionally more prevalent among JRTs in Japan, and it is possible that variant carriers remain hidden in other countries. Owing to the lower frequency of the germline *APC* variant in JRTs, the disease may not have attracted much attention in other countries. Therefore, it is necessary for veterinary clinicians and pathologists worldwide to raise awareness of this novel hereditary disease.

In the present study, despite the relatively limited number of examined cases, the germline *APC* variant was not detected in any dogs with GI cancer in any breeds other than JRTs, suggesting that the variant is specific for the JRT breed. In dogs, although many causative variants are breed-specific, several variants, such as those underlying degenerative myelopathy or hyperuricosuria, are common to multiple breeds [1, 13, 14, 21, 22]. Such variants are thought to be ancient in origin and are therefore potentially widespread across breeds. The absence of the germline *APC* variant in breeds other than JRTs implies that the variant arose *de novo* in a JRT after the establishment of this dog breed.

In veterinary research, it is often difficult to collect sufficient high-quality genomic DNA samples from animals with rare disorders. In the present study, we overcame the low incidence of GI epithelial tumors in dogs [2, 3] by utilizing DNA samples stored in the canine genome bank recently established in Japan. As shown in a previous study [23], canine biobanks will assist in the discovery of novel hereditary disorders by validating the association between identified germline variants and specific diseases. In fact, JRTs with germline *APC* variant have been found in dogs with GI cancers in the canine genome bank.

Another purpose of the present study was to verify the utility and detection limits of PCR-based genotyping assays established in our previous study [9]. In this study, the PCR-RFLP assay successfully determined the *APC* genotypes of all the examined JRTs with blood-derived genomic DNA samples, demonstrating the practical usefulness of this assay. In fact, this assay has been put into practical use in Japan and is offered by a commercial laboratory as DNA testing for hereditary GI polyposis. In contrast, the retrospective analysis revealed that it would be difficult to always make a precise diagnosis from FFPE samples using only the PCR-RFLP assay because of the difficulty in obtaining high-quality genomic DNA from FFPE samples. In such cases, as shown in the present study, the combined use of PCR-direct sequencing can compensate for the defect.

## Conclusion

In the present study, an epidemiological survey revealed that the current frequency of the germline *APC* variant associated with hereditary GI polyposis in JRTs in Japan was 1.89%, and the frequency remained roughly flat during the last 15 years. Proper genetic screening of breeding animals will prevent transmission of the germline *APC* variant to future generations, which will substantially reduce the prevalence and eventually eradicate this hereditary disease. In addition, the germline *APC* variant was not detected in any dogs with GI epithelial tumors of other breeds; therefore, this form of hereditary GI polyposis is most likely specific to JRTs.

## Methods

### Sample collection and preparation

A total of 863 dogs without any overlap were investigated in this study. Supplementary Table 1 shows individual data of all examined dogs.

#### JRTs

Peripheral blood was collected from 792 JRTs at 93 veterinary clinics in Japan between March and June 2020 (Supplementary Table 1). In Japan, during spring and early summer, dogs undergo annual blood tests for filarial infection before starting anti-filarial agents. When blood was collected from JRTs for this purpose, an additional 500 µL was withdrawn for the present study with the consent of dog owners. All JRTs who visited the veterinary clinics within the study period, irrespective of their current and previous medical history, were included in the present study. There was no overlap with JRTs that were investigated in our previous study [6]. Blood samples were anticoagulated with EDTA and stored at -20 °C until DNA extraction. Genomic DNA was extracted from the blood samples using a DNeasy Blood & Tissue Kit (QIAGEN, Venlo, Netherlands). The concentration and purity (A260/A280 ratio) of the extracted DNA were measured using a NanoDrop™ Lite spectrophotometer (Thermo Fisher Scientific, Waltham, MA, USA). Concentrations of the extracted genomic DNA ranged widely from 2.2 to 489.0 ng/µL.

#### Dogs with GI tumors of multiple breeds

##### FFPE samples

Archival FFPE specimens, which were diagnosed as adenomas or adenocarcinomas of the GI tract in dogs between 2014 and 2018 at the Laboratory of Veterinary Pathology, Gifu University, were used (Table 4 and Supplementary Table 1). Genomic DNA was extracted from normal tissues on FFPE sections using a QIAamp DNA FFPE Tissue Kit (QIAGEN). The concentration and purity of the extracted DNA were measured using NanoDropTM Lite.

##### Samples from canine genome bank

Genomic DNA samples from dogs with GI epithelial tumors were also obtained from the canine genome bank at Azabu University, Kanagawa, Japan. The samples contained 38 dogs from 18 different breeds, including four JRTs, and a mixed-breed dog (Table 4 and Supplementary Table 1). There was no overlap between JRTs in the genome bank and JRTs investigated in the present epidemiological and our previous studies [6], as well as between other dogs in the genome bank and those in the pathological archive.

### Genotyping assays

#### PCR-RFLP assay

PCR-RFLP assay was performed as previously described [9]. Briefly, DNA fragments of 156 bp containing the variant site at nucleotides 462 and 463 in exon 4 of the canine *APC* gene were amplified by PCR using the primers listed in Table 5, and the products were digested with the restriction enzyme *Mse*I (*Rsp*RSII) (Takara Bio, Shiga, Japan). The digested products were separated by electrophoresis on a 15% polyacrylamide gel (SuperSep™ Ace; Fujifilm Wako Pure Chemical Industries, Osaka, Japan) and visualized with a UV transilluminator after staining with ethidium bromide.

**Table 5:** Primers and probes used for PCR-based assays for detection of the germline *APC* variant

		Sequence		Product size (bp)
*Primers used for PCR-direct sequencing*				385
Primers	sense	5’- AGTCCCACCTTCAAAAATCC		
	antisense	5’- AACTAAAAATGCAATTATCTTGAATG	-3’	
*Primers used for PCR-RFLP and PCR-direct sequencing of FFPE samples*			-3’	156
Primers	sense	5’- TCTTTTGGCATTGTGTAAACTTG		
	antisense	5’- CTTACATTTTCAGTTAAAGGGAGACT	-3’	
*Primers used and probes for TaqMan realtime PCR of genomic bank samples*			-3’	78
Primers	sense	5’- TTTAGTGAGATTCTGAAGTTGAGCATAATA	-3’	
	Antisense	5’- TCATTACTTCTTGCTGATCTTGACAA wild-type allele	-3’	
		5’- (VIC)- CAATCTTTTTCCTTTTC-(MGB)	-3’	
	Probes mutant allele	5’- (FAM)-CAATCTTAATCCTTTTC-(MGB)	-3’	

*VIC* 2-chloro-7’phenyl-1,4-dichloro-6-carboxy-fluorescein, *FAM* 6-carboxyfluorescein, *MGB* minor groove binder

#### PCR-direct sequencing

PCR-direct sequencing was performed as previously described [6, 9]. PCR amplification was performed to amplify a 385-bp fragment containing the entire exon 4 of the canine *APC* gene from the blood samples of JRTs, as well as a 156-bp fragment containing a variant site in exon 4 from the FFPE samples and canine genome bank samples, as previously reported [6]. The primers used are listed in Table 5. After PCR amplification, the purified PCR products were subjected to sequencing analysis using an ABI Prism 3500 Genetic Analyzer (Applied Biosystems, Foster City, CA, USA) with the Big Dye Terminator v3.1 Cycle Sequencing Kit (Thermo Fisher Scientific).

#### TaqMan duplex real-time PCR assay

Real-time PCR with TaqMan probe was performed as previously described [9]. The primers and probes used are listed in Table 5. The ABI StepOnePlus system (Applied Biosystems) was used to amplify and quantify the PCR products. Data were analyzed using StepOne software v2.3 (Applied Biosystems). Allelic type was determined based on a real-time amplification plot constructed based on FAM and VIC fluorescence intensities monitored during each PCR cycle, as well as an allelic discrimination plot constructed based on the signal intensity ratio of FAM and VIC at the end points of PCR amplification.

### Statistical analysis

For statistical analysis, the chi-square test was used to compare the frequency between/among the groups. *P* < 0.05 was considered statistically significant.

## Supplementary Information

**Additional file 1:** **Supplementary Fig. 1.** Full-length gel images of PCR-RFLP assay. Result of polyacrylamide gel electrophoresis of *Mse*I-digested PCR products amplified from blood samples (A), FFPE samples (B) and genome bank samples (C). Synthetic wild-type and variant DNA and DNA samples of previously detected carrier and non-carrier JRTs are used as controls. (A) Representative image of PCR-RFLP assay using blood samples of JRTs. Case nos. JRT 317 and JRT 321 are determined to be APC variant carriers based on the presence of bands at 51 and 57 bp derivedfrom APC variant allele. (B) PCR-RFLP assay using FFPE samples. In the analysis,it is impossible to recognize the clear fragment pattern in some lanes. Three fulllength images of three different gels are grouped. (C) PCR-RFLP assay using genomebank samples. In the analysis, three cases, case nos. GB018, GB033 and GB039, are determined to be APC variant carriers based on the presence of bands at 51 and 57bp derived from APC variant allele. Three full length images of three differentgels are grouped. WT, wild-type; MT, mutant type; N, non-carrier; C, carrier, DW, distilled water.

**Additional file 2: Supplementary Fig. 2.** PCR-direct sequencing.(A)Representative result of a carrier JRT, case no. JRT 221. DNA sequencing of PCR-amplified 385-bp fragment containing entire exon 4 of the canine APC gene. The red arrows indicate 2-bp substitution at codons 154 and 155. (B) Representative result of a FFPE sample, case no. FFPE032. DNA sequencing of PCR-amplified 156-bp fragment containingthe variant site in exon4. The APC variant at codons 154 and 155 is absent. The black arrows indicate variant sites at codons 154 and 155.

**Additional file 3:** **Supplementary Table 1.** Case information. 

## Data Availability

The datasets used and/or analyzed during the current study are available from the corresponding author upon reasonable request. DNA sequence data of the germline *APC* variant is available in GenBank repository (ID: LC706270-LC706287).

## Abbreviations

APC: Adenomatous polyposis coli
EDTA: Ethylenediaminetetraacetic acid
GI: Gastrointestinal
JRT: Jack Russell Terrier
FFPE: Formalin-fixed paraffin-embedded
PCR: Polymerase chain reaction
PCR-RFLP: PCR-restriction fragment length polymorphism
