# Supplementary material for: Molecular epidemiological study of germline APC variant associated with hereditary gastrointestinal polyposis in dogs: current frequency in Jack Russell Terriers in Japan and breed distribution
Source: BMC Vet Res. 2022 Jun 18;18:230. doi: 10.1186/s12917-022-03338-w (PMC9206296; doi:10.1186/s12917-022-03338-w)
Supplement: Supplementary file 1 — Additional file 1: Supplementary Fig. 1. Full-length gel images of PCR-RFLP assay. Result of polyacrylamide gel electrophoresis of MseI-digested PCR products amplified from blood samples (A), FFPE samples (B) and genome bank samples (C). Synthetic wild-type and variant DNA and DNA samples of previously detected carrier and non-carrier JRTs are used as controls. (A) Representative image of PCR-RFLP assay using blood samples of JRTs. Case nos. JRT 317 and JRT 321 are determined to be APC variant carriers based on the presence of bands at 51 and 57 bp derivedfrom APC variant allele. (B) PCR-RFLP assay using FFPE samples. In the analysis,it is impossible to recognize the clear fragment pattern in some lanes. Three fulllength images of three different gels are grouped. (C) PCR-RFLP assay using genomebank samples. In the analysis, three cases, case nos. GB018, GB033 and GB039, are determined to be APC variant carriers based on the presence of bands at 51 and 57bp derived from APC variant allele. Three full length images of three differentgels are grouped. WT, wild-type; MT, mutant type; N, non-carrier; C, carrier, DW, distilled water. [file 12917_2022_3338_MOESM1_ESM.pdf]

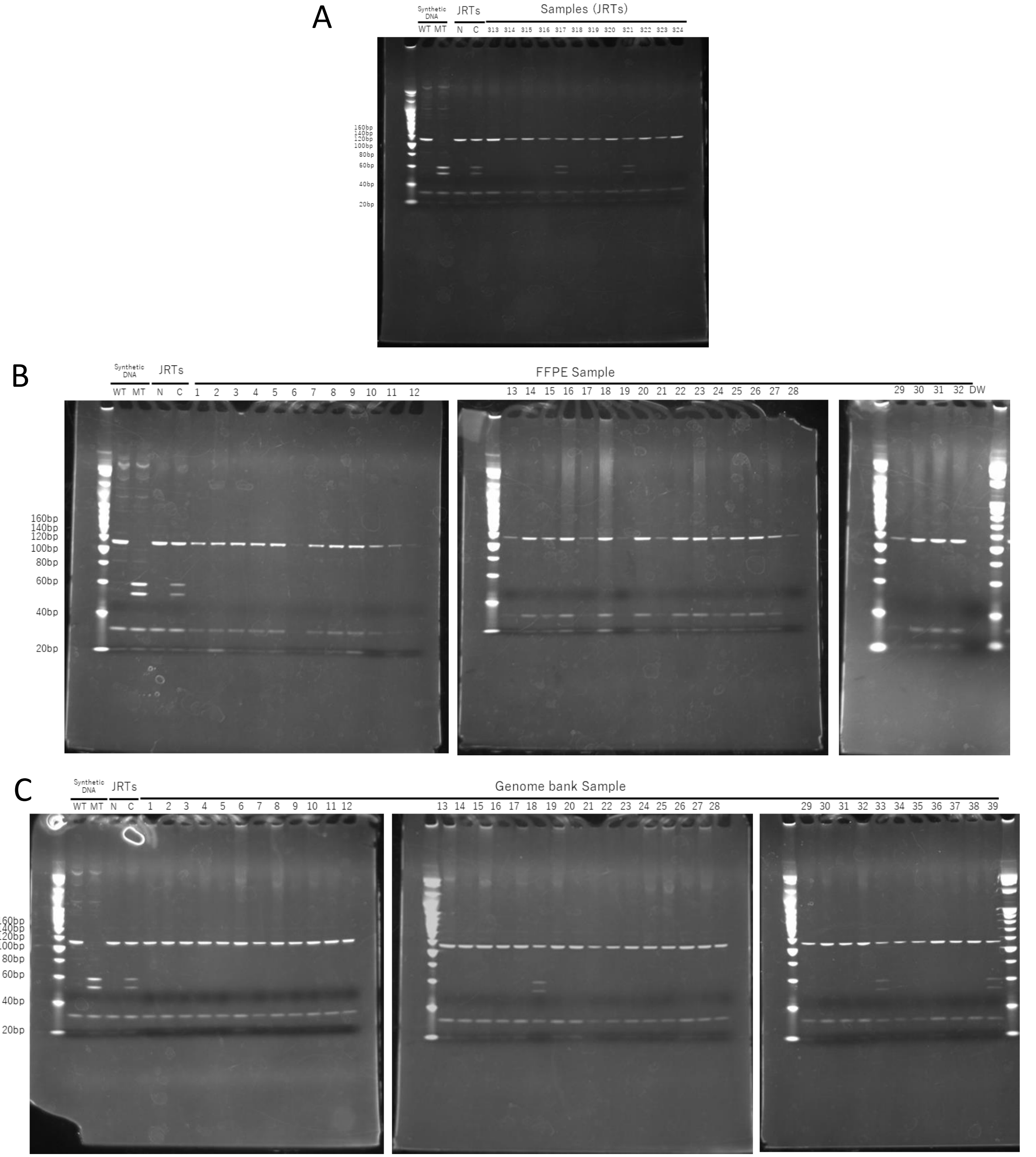

Supplementary Fig. 1. Full-length gel images of PCR-RFLP assay.

Result of polyacrylamide gel electrophoresis of *Mse*I-digested PCR products amplified from blood samples (A), FFPE samples (B) and genome bank samples (C). Synthetic wild-type and variant DNA and DNA samples of previously detected carrier and non-carrier JRTs are used as controls. (A) Representative image of PCR-RFLP assay using blood samples of JRTs. Case nos. JRT317 and JRT321 are determined to be *APC* variant carriers based on the presence of bands at 51 and 57 bp derived from *APC* variant allele. (B) PCR-RFLP assay using FFPE samples. In the analysis, it is impossible to recognize the clear fragment pattern in some lanes. Three full length images of three different gels are grouped. (C) PCR-RFLP assay using genome bank samples. In the analysis, three cases, case nos. GB018, GB033 and GB039, are determined to be *APC* variant carriers based on the presence of bands at 51 and 57 bp derived from *APC* variant allele. Three full length images of three different gels are grouped. WT, wild-type; MT, mutant type; N, non-carrier; C, carrier, DW, distilled water.
