# Supplementary material for: Molecular epidemiological study of germline APC variant associated with hereditary gastrointestinal polyposis in dogs: current frequency in Jack Russell Terriers in Japan and breed distribution
Source: BMC Vet Res. 2022 Jun 18;18:230. doi: 10.1186/s12917-022-03338-w (PMC9206296; doi:10.1186/s12917-022-03338-w)
Supplement: Supplementary file 2 — Additional file 2: Supplementary Fig. 2. PCR-direct sequencing.(A)Representative result of a carrier JRT, case no. JRT 221. DNA sequencing of PCR-amplified 385-bp fragment containing entire exon 4 of the canine APC gene. The red arrows indicate 2-bp substitution at codons 154 and 155. (B) Representative result of a FFPE sample, case no. FFPE032. DNA sequencing of PCR-amplified 156-bp fragment containingthe variant site in exon4. The APC variant at codons 154 and 155 is absent. The black arrows indicate variant sites at codons 154 and 155. [file 12917_2022_3338_MOESM2_ESM.pdf]

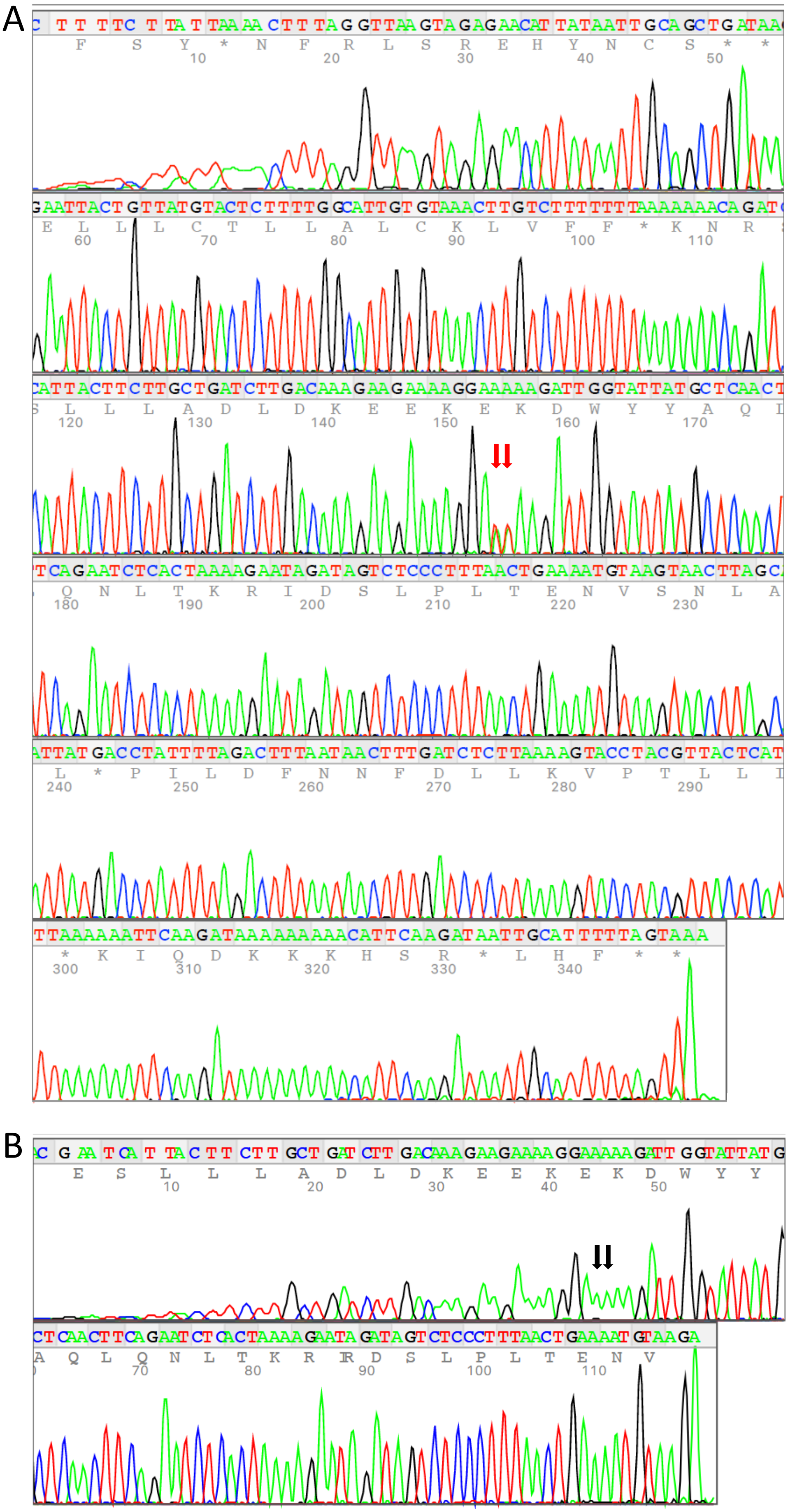

Supplementary Fig. 2. PCR-direct sequencing.

(A) Representative result of a carrier JRT, case no. JRT221. DNA sequencing of PCR-amplified 385-bp fragment containing entire exon 4 of the canine *APC* gene. The red arrows indicate 2-bp substitution at codons 154 and 155. (B) Representative result of a FFPE sample, case no. FFPE032. DNA sequencing of PCR-amplified 156-bp fragment containing the variant site in exon 4. The *APC* variant at codons 154 and 155 is absent. The black arrows indicate variant sites at codons 154 and 155.
