# Supplementary material for: Molecular epidemiological study of germline APC variant associated with hereditary gastrointestinal polyposis in dogs: current frequency in Jack Russell Terriers in Japan and breed distribution
Source: BMC Vet Res. 2022 Jun 18;18:230. doi: 10.1186/s12917-022-03338-w (PMC9206296; doi:10.1186/s12917-022-03338-w)
Supplement: Supplementary file 3 — Additional file 3: Supplementary Table 1. Case information. [file 12917_2022_3338_MOESM3_ESM.pdf]

Supplementary Table 1. Characteristics information and *APC* variant status of samples.

Signalment of Jack Russell terriers examine in prevalence survey of hereditary gastrointestinal polyposis

Case No.	Birth year	Sex	Dog breed	Coat type	*APC* variant status
JRT001	2018	Male	Jack Russell Terrier	rough	Non-carrier
JRT002	2014	Male	Jack Russell Terrier	rough	Non-carrier
JRT003	2015	Male	Jack Russell Terrier	rough	Non-carrier
JRT004	2011	Male	Jack Russell Terrier	rough	Non-carrier
JRT005	2017	Male	Jack Russell Terrier	rough	Non-carrier
JRT006	2016	Male	Jack Russell Terrier	rough	Non-carrier
JRT007	2006	Male (castrated)	Jack Russell Terrier	rough	Non-carrier
JRT008	2009	Male (castrated)	Jack Russell Terrier	broken	Non-carrier
JRT009	2015	Male (castrated)	Jack Russell Terrier	smooth	Non-carrier
JRT010	2007	Male (castrated)	Jack Russell Terrier	broken	Non-carrier
JRT011	2013	Female (spayed)	Jack Russell Terrier	smooth	Non-carrier
JRT012	2010	Female (spayed)	Jack Russell Terrier	broken	Non-carrier
JRT013	2008	Female (spayed)	Jack Russell Terrier	smooth	Non-carrier
JRT014	2012	Female (spayed)	Jack Russell Terrier	broken	Non-carrier
JRT015	2006	Female (spayed)	Jack Russell Terrier	broken	Non-carrier
JRT016	2010	Female (spayed)	Jack Russell Terrier	broken	Non-carrier
JRT017	2014	Male (castrated)	Jack Russell Terrier	rough	Non-carrier
JRT018	2004	Female (spayed)	Jack Russell Terrier	smooth	Non-carrier
JRT019	2017	Male (castrated)	Jack Russell Terrier	N.D.	Non-carrier
JRT020	2014	Male	Jack Russell Terrier	N.D.	Non-carrier
JRT021	2008	Female (spayed)	Jack Russell Terrier	smooth	Non-carrier
JRT022	2015	Male (castrated)	Jack Russell Terrier	broken	Non-carrier
JRT023	2014	Female (spayed)	Jack Russell Terrier	rough	Non-carrier
JRT024	2019	Female	Jack Russell Terrier	smooth	Non-carrier
JRT025	2014	Male (castrated)	Jack Russell Terrier	broken	Non-carrier
JRT026	2004	Male (castrated)	Jack Russell Terrier	smooth	Non-carrier
JRT027	2013	Female	Jack Russell Terrier	N.D.	Non-carrier
JRT028	2018	Male (castrated)	Jack Russell Terrier	smooth	Non-carrier
JRT029	2013	Male (castrated)	Jack Russell Terrier	broken	Non-carrier
JRT030	2017	Female (spayed)	Jack Russell Terrier	smooth	Non-carrier
JRT031	2010	Female (spayed)	Jack Russell Terrier	rough	Non-carrier
JRT032	2005	Male (castrated)	Jack Russell Terrier	broken	Non-carrier
JRT033	2011	Male (castrated)	Jack Russell Terrier	rough	Non-carrier
JRT034	2011	Male (castrated)	Jack Russell Terrier	smooth	Non-carrier
JRT035	2007	Female (spayed)	Jack Russell Terrier	broken	Non-carrier
JRT036	2017	Male (castrated)	Jack Russell Terrier	smooth	Non-carrier
JRT037	2010	Female (spayed)	Jack Russell Terrier	smooth	Non-carrier
JRT038	2009	Male	Jack Russell Terrier	rough	Non-carrier
JRT039	2016	Female (spayed)	Jack Russell Terrier	broken	Non-carrier
JRT040	2006	Male	Jack Russell Terrier	smooth	Non-carrier
JRT041	2013	Male	Jack Russell Terrier	smooth	Non-carrier
JRT042	2009	Male	Jack Russell Terrier	N.D.	Non-carrier
JRT043	2016	Male	Jack Russell Terrier	N.D.	Non-carrier
JRT044	2009	Female (spayed)	Jack Russell Terrier	broken	Non-carrier
JRT045	2009	Female (spayed)	Jack Russell Terrier	N.D.	Non-carrier
JRT046	2019	Male (castrated)	Jack Russell Terrier	N.D.	Non-carrier
JRT047	2008	Male (castrated)	Jack Russell Terrier	broken	Non-carrier
JRT048	2006	Female (spayed)	Jack Russell Terrier	broken	Non-carrier
JRT049	2018	Female (spayed)	Jack Russell Terrier	smooth	Non-carrier
JRT050	2011	Male	Jack Russell Terrier	rough	Non-carrier
JRT051	2011	Male (castrated)	Jack Russell Terrier	rough	Non-carrier
JRT052	2011	Male	Jack Russell Terrier	broken	Non-carrier
JRT053	2013	Female (spayed)	Jack Russell Terrier	smooth	Non-carrier
JRT054	2010	Female (spayed)	Jack Russell Terrier	smooth	Non-carrier
JRT055	2010	Male	Jack Russell Terrier	smooth	Carrier
JRT056	2010	Male	Jack Russell Terrier	broken	Non-carrier
JRT057	2012	Male (castrated)	Jack Russell Terrier	smooth	Non-carrier
JRT058	2011	Male (castrated)	Jack Russell Terrier	smooth	Non-carrier
JRT059	2014	Female (spayed)	Jack Russell Terrier	broken	Non-carrier
JRT060	2010	Female (spayed)	Jack Russell Terrier	rough	Non-carrier
JRT061	2001	Female (spayed)	Jack Russell Terrier	broken	Non-carrier
JRT062	2006	Female (spayed)	Jack Russell Terrier	rough	Non-carrier
JRT063	2013	Female (spayed)	Jack Russell Terrier	smooth	Non-carrier
JRT064	2012	Female (spayed)	Jack Russell Terrier	rough	Non-carrier
JRT065	2006	Female (spayed)	Jack Russell Terrier	broken	Non-carrier
JRT066	2009	Female (spayed)	Jack Russell Terrier	smooth	Non-carrier
JRT067	2015	Female (spayed)	Jack Russell Terrier	rough	Non-carrier
JRT068	2013	Female (spayed)	Jack Russell Terrier	rough	Non-carrier
JRT069	2011	Male (castrated)	Jack Russell Terrier	smooth	Non-carrier
JRT070	2008	Female (spayed)	Jack Russell Terrier	broken	Non-carrier
JRT071	2013	Female (spayed)	Jack Russell Terrier	broken	Non-carrier
JRT072	2010	Female (spayed)	Jack Russell Terrier	smooth	Non-carrier
JRT073	2003	Female (spayed)	Jack Russell Terrier	broken	Non-carrier
JRT074	2014	Male (castrated)	Jack Russell Terrier	broken	Non-carrier
JRT075	2018	Male (castrated)	Jack Russell Terrier	broken	Non-carrier
JRT076	2018	Female (spayed)	Jack Russell Terrier	broken	Non-carrier

JRT077	2012	Male (castrated)	Jack Russell Terrier	broken	Non-carrier
JRT078	2009	Male	Jack Russell Terrier	smooth	Non-carrier
JRT079	2015	Male (castrated)	Jack Russell Terrier	smooth	Non-carrier
JRT080	2004	Female	Jack Russell Terrier	smooth	Non-carrier
JRT081	2007	Female (spayed)	Jack Russell Terrier	smooth	Non-carrier
JRT082	2003	Female (spayed)	Jack Russell Terrier	smooth	Non-carrier
JRT083	2011	Male	Jack Russell Terrier	rough	Non-carrier
JRT084	2019	Female (spayed)	Jack Russell Terrier	smooth	Non-carrier
JRT085	2013	Female (spayed)	Jack Russell Terrier	broken	Non-carrier
JRT086	2006	Female (spayed)	Jack Russell Terrier	rough	Non-carrier
JRT087	2014	Male (castrated)	Jack Russell Terrier	smooth	Non-carrier
JRT088	2013	Male (castrated)	Jack Russell Terrier	rough	Non-carrier
JRT089	2011	Male	Jack Russell Terrier	broken	Non-carrier
JRT090	2015	Male	Jack Russell Terrier	smooth	Non-carrier
JRT091	2010	Female (spayed)	Jack Russell Terrier	rough	Non-carrier
JRT092	2006	Female (spayed)	Jack Russell Terrier	smooth	Non-carrier
JRT093	2006	Male	Jack Russell Terrier	smooth	Non-carrier
JRT094	2007	Female (spayed)	Jack Russell Terrier	broken	Non-carrier
JRT095	2005	Female (spayed)	Jack Russell Terrier	smooth	Non-carrier
JRT096	2014	Male (castrated)	Jack Russell Terrier	smooth	Non-carrier
JRT097	2014	Male (castrated)	Jack Russell Terrier	smooth	Non-carrier
JRT098	2006	Male (castrated)	Jack Russell Terrier	smooth	Non-carrier
JRT099	2008	Female (spayed)	Jack Russell Terrier	smooth	Non-carrier
JRT100	2005	Male (castrated)	Jack Russell Terrier	smooth	Non-carrier
JRT101	2003	Male (castrated)	Jack Russell Terrier	smooth	Non-carrier
JRT102	2007	Male (castrated)	Jack Russell Terrier	smooth	Non-carrier
JRT103	2011	Female (spayed)	Jack Russell Terrier	smooth	Non-carrier
JRT104	2014	Male (castrated)	Jack Russell Terrier	smooth	Non-carrier
JRT105	2016	Female (spayed)	Jack Russell Terrier	smooth	Non-carrier
JRT106	2016	Male	Jack Russell Terrier	smooth	Non-carrier
JRT107	2006	Female (spayed)	Jack Russell Terrier	broken	Non-carrier
JRT108	2012	Male (castrated)	Jack Russell Terrier	smooth	Non-carrier
JRT109	2004	Male	Jack Russell Terrier	broken	Non-carrier
JRT110	2011	Female (spayed)	Jack Russell Terrier	smooth	Non-carrier
JRT111	2008	Male (castrated)	Jack Russell Terrier	broken	Non-carrier
JRT112	2015	Male (castrated)	Jack Russell Terrier	broken	Non-carrier
JRT113	2016	Female (spayed)	Jack Russell Terrier	broken	Non-carrier
JRT114	2015	Female (spayed)	Jack Russell Terrier	smooth	Non-carrier
JRT115	2011	Female (spayed)	Jack Russell Terrier	smooth	Non-carrier
JRT116	2010	Male (castrated)	Jack Russell Terrier	broken	Non-carrier
JRT117	2013	Female	Jack Russell Terrier	smooth	Non-carrier
JRT118	2013	Female	Jack Russell Terrier	broken	Non-carrier
JRT119	2007	Female	Jack Russell Terrier	smooth	Non-carrier
JRT120	2011	Female (spayed)	Jack Russell Terrier	N.D.	Non-carrier
JRT121	2013	Female (spayed)	Jack Russell Terrier	smooth	Non-carrier
JRT122	2017	Female (spayed)	Jack Russell Terrier	smooth	Non-carrier
JRT123	2009	Female (spayed)	Jack Russell Terrier	smooth	Carrier
JRT124	2005	Female	Jack Russell Terrier	smooth	Non-carrier
JRT125	2009	Male (castrated)	Jack Russell Terrier	rough	Non-carrier
JRT126	2011	Male (castrated)	Jack Russell Terrier	smooth	Non-carrier
JRT127	2013	Male (castrated)	Jack Russell Terrier	smooth	Non-carrier
JRT128	2006	Male	Jack Russell Terrier	smooth	Non-carrier
JRT129	2019	Male	Jack Russell Terrier	smooth	Non-carrier
JRT130	2010	Male (castrated)	Jack Russell Terrier	smooth	Non-carrier
JRT131	2008	Male	Jack Russell Terrier	smooth	Non-carrier
JRT132	2018	Female	Jack Russell Terrier	N.D.	Non-carrier
JRT133	2004	Male (castrated)	Jack Russell Terrier	broken	Non-carrier
JRT134	2015	Male (castrated)	Jack Russell Terrier	smooth	Non-carrier
JRT135	2017	Female (spayed)	Jack Russell Terrier	broken	Non-carrier
JRT136	2005	Female (spayed)	Jack Russell Terrier	smooth	Non-carrier
JRT137	2019	Male (castrated)	Jack Russell Terrier	broken	Non-carrier
JRT138	2009	Male (castrated)	Jack Russell Terrier	broken	Non-carrier
JRT139	2006	Male (castrated)	Jack Russell Terrier	smooth	Non-carrier
JRT140	2010	Female	Jack Russell Terrier	smooth	Non-carrier
JRT141	2006	Male	Jack Russell Terrier	broken	Non-carrier
JRT142	2019	Female (spayed)	Jack Russell Terrier	smooth	Non-carrier
JRT143	2008	Male	Jack Russell Terrier	smooth	Non-carrier
JRT144	2008	Male	Jack Russell Terrier	broken	Non-carrier
JRT145	2014	Female (spayed)	Jack Russell Terrier	smooth	Non-carrier
JRT146	2009	Female (spayed)	Jack Russell Terrier	broken	Non-carrier
JRT147	2018	Male (castrated)	Jack Russell Terrier	smooth	Non-carrier
JRT148	2016	Female (spayed)	Jack Russell Terrier	smooth	Non-carrier
JRT149	2013	Female	Jack Russell Terrier	rough	Non-carrier
JRT150	2009	Male (castrated)	Jack Russell Terrier	broken	Non-carrier
JRT151	2011	Male (castrated)	Jack Russell Terrier	smooth	Non-carrier
JRT152	2012	Female	Jack Russell Terrier	smooth	Carrier
JRT153	2016	Male (castrated)	Jack Russell Terrier	smooth	Non-carrier
JRT154	2019	Male (castrated)	Jack Russell Terrier	N.D.	Non-carrier
JRT155	2013	Female (spayed)	Jack Russell Terrier	smooth	Non-carrier
JRT156	2015	Female (spayed)	Jack Russell Terrier	broken	Non-carrier
JRT157	2013	Male (castrated)	Jack Russell Terrier	broken	Non-carrier
JRT158	2014	Female	Jack Russell Terrier	rough	Non-carrier

JRT159	2014	Female (spayed)	Jack Russell Terrier	smooth	Non-carrier
JRT160	2008	Female (spayed)	Jack Russell Terrier	broken	Non-carrier
JRT161	2012	Male (castrated)	Jack Russell Terrier	broken	Non-carrier
JRT162	2004	Female (spayed)	Jack Russell Terrier	smooth	Non-carrier
JRT163	2005	Male (castrated)	Jack Russell Terrier	rough	Non-carrier
JRT164	2004	Male	Jack Russell Terrier	smooth	Non-carrier
JRT165	2013	Male (castrated)	Jack Russell Terrier	smooth	Non-carrier
JRT166	2012	Female (spayed)	Jack Russell Terrier	smooth	Non-carrier
JRT167	2008	Male (castrated)	Jack Russell Terrier	smooth	Non-carrier
JRT168	2019	Female	Jack Russell Terrier	smooth	Non-carrier
JRT169	2008	Male (castrated)	Jack Russell Terrier	smooth	Non-carrier
JRT170	2013	Female (spayed)	Jack Russell Terrier	smooth	Non-carrier
JRT171	2017	Female (spayed)	Jack Russell Terrier	smooth	Non-carrier
JRT172	2010	Male	Jack Russell Terrier	broken	Non-carrier
JRT173	2010	Male	Jack Russell Terrier	smooth	Non-carrier
JRT174	2006	Female (spayed)	Jack Russell Terrier	smooth	Non-carrier
JRT175	2009	Male (castrated)	Jack Russell Terrier	smooth	Non-carrier
JRT176	2011	Male	Jack Russell Terrier	smooth	Non-carrier
JRT177	2010	Female (spayed)	Jack Russell Terrier	broken	Non-carrier
JRT178	2012	Male (castrated)	Jack Russell Terrier	smooth	Non-carrier
JRT179	2005	Female (spayed)	Jack Russell Terrier	N.D.	Non-carrier
JRT180	2009	Female (spayed)	Jack Russell Terrier	smooth	Non-carrier
JRT181	2017	Male (castrated)	Jack Russell Terrier	N.D.	Non-carrier
JRT182	2014	Male (castrated)	Jack Russell Terrier	N.D.	Non-carrier
JRT183	2008	Female (spayed)	Jack Russell Terrier	N.D.	Non-carrier
JRT184	2005	Male (castrated)	Jack Russell Terrier	N.D.	Non-carrier
JRT185	2002	Male (castrated)	Jack Russell Terrier	N.D.	Non-carrier
JRT186	2011	Male (castrated)	Jack Russell Terrier	N.D.	Non-carrier
JRT187	2010	Male (castrated)	Jack Russell Terrier	rough	Non-carrier
JRT188	2005	Female (spayed)	Jack Russell Terrier	N.D.	Non-carrier
JRT189	2008	Female (spayed)	Jack Russell Terrier	N.D.	Non-carrier
JRT190	2012	Female	Jack Russell Terrier	broken	Non-carrier
JRT191	2011	Female	Jack Russell Terrier	rough	Non-carrier
JRT192	2010	Male	Jack Russell Terrier	smooth	Non-carrier
JRT193	2011	Male	Jack Russell Terrier	smooth	Non-carrier
JRT194	2014	Male (castrated)	Jack Russell Terrier	smooth	Non-carrier
JRT195	2019	Female (spayed)	Jack Russell Terrier	smooth	Non-carrier
JRT196	2017	Male	Jack Russell Terrier	smooth	Carrier
JRT197	2011	Male (castrated)	Jack Russell Terrier	smooth	Non-carrier
JRT198	2006	Male (castrated)	Jack Russell Terrier	smooth	Non-carrier
JRT199	2011	Male (castrated)	Jack Russell Terrier	broken	Non-carrier
JRT200	2019	Female	Jack Russell Terrier	smooth	Non-carrier
JRT201	2010	Male (castrated)	Jack Russell Terrier	broken	Non-carrier
JRT202	2012	Male (castrated)	Jack Russell Terrier	broken	Non-carrier
JRT203	2014	Female (spayed)	Jack Russell Terrier	broken	Carrier
JRT204	2007	Female (spayed)	Jack Russell Terrier	rough	Non-carrier
JRT205	2010	Male	Jack Russell Terrier	broken	Non-carrier
JRT206	2004	Female (spayed)	Jack Russell Terrier	smooth	Non-carrier
JRT207	2014	Female (spayed)	Jack Russell Terrier	smooth	Non-carrier
JRT208	2006	Female (spayed)	Jack Russell Terrier	smooth	Non-carrier
JRT209	2011	Female (spayed)	Jack Russell Terrier	smooth	Non-carrier
JRT210	2006	Female (spayed)	Jack Russell Terrier	smooth	Non-carrier
JRT211	2011	Female	Jack Russell Terrier	smooth	Non-carrier
JRT212	2009	Male	Jack Russell Terrier	smooth	Non-carrier
JRT213	2016	Female (spayed)	Jack Russell Terrier	broken	Non-carrier
JRT214	2006	Female	Jack Russell Terrier	rough	Non-carrier
JRT215	2007	Male (castrated)	Jack Russell Terrier	smooth	Non-carrier
JRT216	2013	Male (castrated)	Jack Russell Terrier	smooth	Carrier
JRT217	2015	Female (spayed)	Jack Russell Terrier	smooth	Non-carrier
JRT218	2005	Female (spayed)	Jack Russell Terrier	smooth	Non-carrier
JRT219	2012	Female (spayed)	Jack Russell Terrier	rough	Non-carrier
JRT220	2017	Female (spayed)	Jack Russell Terrier	N.D.	Non-carrier
JRT221	2016	Female (spayed)	Jack Russell Terrier	smooth	Carrier
JRT222	2005	Male (castrated)	Jack Russell Terrier	smooth	Non-carrier
JRT223	2005	Male (castrated)	Jack Russell Terrier	smooth	Non-carrier
JRT224	2013	Female	Jack Russell Terrier	smooth	Non-carrier
JRT225	2015	Female (spayed)	Jack Russell Terrier	broken	Non-carrier
JRT226	2006	Male (castrated)	Jack Russell Terrier	rough	Non-carrier
JRT227	2013	Male (castrated)	Jack Russell Terrier	broken	Non-carrier
JRT228	2012	Female (spayed)	Jack Russell Terrier	smooth	Non-carrier
JRT229	2006	Male (castrated)	Jack Russell Terrier	smooth	Non-carrier
JRT230	2012	Male	Jack Russell Terrier	broken	Non-carrier
JRT231	2005	Female (spayed)	Jack Russell Terrier	broken	Non-carrier
JRT232	2015	Male (castrated)	Jack Russell Terrier	broken	Non-carrier
JRT233	2019	Male	Jack Russell Terrier	smooth	Non-carrier
JRT234	2004	Male (castrated)	Jack Russell Terrier	N.D.	Non-carrier
JRT235	2016	Female (spayed)	Jack Russell Terrier	broken	Non-carrier
JRT236	2005	Male (castrated)	Jack Russell Terrier	smooth	Non-carrier
JRT237	2015	Female	Jack Russell Terrier	smooth	Non-carrier
JRT238	2012	Female (spayed)	Jack Russell Terrier	smooth	Non-carrier
JRT239	2019	Female (spayed)	Jack Russell Terrier	N.D.	Non-carrier
JRT240	2018	Male (castrated)	Jack Russell Terrier	broken	Non-carrier

JRT241	2008	Female (spayed)	Jack Russell Terrier	broken	Non-carrier
JRT242	2016	Male (castrated)	Jack Russell Terrier	broken	Non-carrier
JRT243	2010	Female (spayed)	Jack Russell Terrier	smooth	Non-carrier
JRT244	2008	Female (spayed)	Jack Russell Terrier	smooth	Non-carrier
JRT245	2011	Male (castrated)	Jack Russell Terrier	broken	Non-carrier
JRT246	2013	Female (spayed)	Jack Russell Terrier	smooth	Non-carrier
JRT247	2007	Female (spayed)	Jack Russell Terrier	smooth	Non-carrier
JRT248	2015	Female (spayed)	Jack Russell Terrier	smooth	Non-carrier
JRT249	2010	Male	Jack Russell Terrier	rough	Non-carrier
JRT250	2015	Male (castrated)	Jack Russell Terrier	broken	Non-carrier
JRT251	2011	Female (spayed)	Jack Russell Terrier	smooth	Non-carrier
JRT252	2015	Female (spayed)	Jack Russell Terrier	smooth	Non-carrier
JRT253	2014	Male	Jack Russell Terrier	broken	Non-carrier
JRT254	2012	Female (spayed)	Jack Russell Terrier	rough	Non-carrier
JRT255	2007	Male	Jack Russell Terrier	rough	Non-carrier
JRT256	2007	Male (unspecified)	Jack Russell Terrier	broken	Non-carrier
JRT257	2007	Female (spayed)	Jack Russell Terrier	broken	Non-carrier
JRT258	2011	Female (spayed)	Jack Russell Terrier	smooth	Non-carrier
JRT259	2013	Male (castrated)	Jack Russell Terrier	rough	Non-carrier
JRT260	2013	Male (castrated)	Jack Russell Terrier	broken	Non-carrier
JRT261	2010	Male (castrated)	Jack Russell Terrier	smooth	Non-carrier
JRT262	2012	Female (spayed)	Jack Russell Terrier	broken	Non-carrier
JRT263	2012	Male (castrated)	Jack Russell Terrier	rough	Non-carrier
JRT264	2014	Male (castrated)	Jack Russell Terrier	rough	Carrier
JRT265	2009	Male	Jack Russell Terrier	smooth	Non-carrier
JRT266	2008	Female (spayed)	Jack Russell Terrier	rough	Non-carrier
JRT267	2003	Female (spayed)	Jack Russell Terrier	rough	Non-carrier
JRT268	2014	Male (castrated)	Jack Russell Terrier	broken	Non-carrier
JRT269	2010	Female	Jack Russell Terrier	broken	Non-carrier
JRT270	2010	Male	Jack Russell Terrier	smooth	Non-carrier
JRT271	2014	Male (castrated)	Jack Russell Terrier	broken	Non-carrier
JRT272	2014	Female (spayed)	Jack Russell Terrier	broken	Non-carrier
JRT273	2016	Female	Jack Russell Terrier	rough	Non-carrier
JRT274	2010	Male (castrated)	Jack Russell Terrier	rough	Non-carrier
JRT275	2015	Female (spayed)	Jack Russell Terrier	smooth	Non-carrier
JRT276	2016	Male	Jack Russell Terrier	N.D.	Non-carrier
JRT277	2014	Male (castrated)	Jack Russell Terrier	rough	Non-carrier
JRT278	2009	Male (castrated)	Jack Russell Terrier	rough	Non-carrier
JRT279	2004	Male (castrated)	Jack Russell Terrier	rough	Non-carrier
JRT280	2007	Female (spayed)	Jack Russell Terrier	rough	Non-carrier
JRT281	2016	Male (castrated)	Jack Russell Terrier	smooth	Non-carrier
JRT282	2017	Male (castrated)	Jack Russell Terrier	rough	Non-carrier
JRT283	2010	Female (spayed)	Jack Russell Terrier	smooth	Non-carrier
JRT284	2008	Female	Jack Russell Terrier	broken	Non-carrier
JRT285	2016	Male (castrated)	Jack Russell Terrier	smooth	Non-carrier
JRT286	2015	Female (spayed)	Jack Russell Terrier	rough	Non-carrier
JRT287	2013	Female (spayed)	Jack Russell Terrier	broken	Non-carrier
JRT288	2011	Male	Jack Russell Terrier	smooth	Non-carrier
JRT289	2010	Female (spayed)	Jack Russell Terrier	N.D.	Non-carrier
JRT290	2018	Male	Jack Russell Terrier	smooth	Non-carrier
JRT291	2010	Female (spayed)	Jack Russell Terrier	smooth	Non-carrier
JRT292	2013	Male (castrated)	Jack Russell Terrier	smooth	Non-carrier
JRT293	2015	Female	Jack Russell Terrier	broken	Non-carrier
JRT294	2018	Male	Jack Russell Terrier	rough	Non-carrier
JRT295	2008	Female	Jack Russell Terrier	broken	Non-carrier
JRT296	2011	Male (castrated)	Jack Russell Terrier	rough	Non-carrier
JRT297	2013	Female	Jack Russell Terrier	broken	Non-carrier
JRT298	2017	Female	Jack Russell Terrier	rough	Non-carrier
JRT299	2018	Male (castrated)	Jack Russell Terrier	smooth	Non-carrier
JRT300	2011	Female	Jack Russell Terrier	broken	Non-carrier
JRT301	2012	Male (castrated)	Jack Russell Terrier	smooth	Non-carrier
JRT302	2012	Female (spayed)	Jack Russell Terrier	smooth	Non-carrier
JRT303	2005	Female (spayed)	Jack Russell Terrier	broken	Non-carrier
JRT304	2012	Female (spayed)	Jack Russell Terrier	smooth	Non-carrier
JRT305	2014	Male	Jack Russell Terrier	smooth	Non-carrier
JRT306	2016	Male (castrated)	Jack Russell Terrier	broken	Non-carrier
JRT307	2010	Female (spayed)	Jack Russell Terrier	N.D.	Non-carrier
JRT308	2012	Male	Jack Russell Terrier	rough	Non-carrier
JRT309	2004	Female (spayed)	Jack Russell Terrier	smooth	Non-carrier
JRT310	2013	Male (castrated)	Jack Russell Terrier	rough	Non-carrier
JRT311	2017	Female	Jack Russell Terrier	smooth	Non-carrier
JRT312	2017	Male	Jack Russell Terrier	smooth	Non-carrier
JRT313	2009	Female (spayed)	Jack Russell Terrier	smooth	Non-carrier
JRT314	2007	Female	Jack Russell Terrier	smooth	Non-carrier
JRT315	2010	Male	Jack Russell Terrier	smooth	Non-carrier
JRT316	2017	Female (spayed)	Jack Russell Terrier	smooth	Non-carrier
JRT317	2017	Female (spayed)	Jack Russell Terrier	smooth	Carrier
JRT318	2007	Female	Jack Russell Terrier	smooth	Non-carrier
JRT319	2012	Female (spayed)	Jack Russell Terrier	smooth	Non-carrier
JRT320	2011	Male (castrated)	Jack Russell Terrier	smooth	Non-carrier
JRT321	2009	Female (spayed)	Jack Russell Terrier	broken	Carrier
JRT322	2013	Male	Jack Russell Terrier	smooth	Non-carrier

JRT323	2015	Male	Jack Russell Terrier	broken	Non-carrier
JRT324	2018	Female (spayed)	Jack Russell Terrier	smooth	Non-carrier
JRT325	2008	Female (spayed)	Jack Russell Terrier	broken	Non-carrier
JRT326	2006	Female	Jack Russell Terrier	smooth	Non-carrier
JRT327	2010	Male (castrated)	Jack Russell Terrier	broken	Non-carrier
JRT328	2018	Female (spayed)	Jack Russell Terrier	broken	Non-carrier
JRT329	2009	Male (castrated)	Jack Russell Terrier	smooth	Non-carrier
JRT330	2016	Male (castrated)	Jack Russell Terrier	smooth	Non-carrier
JRT331	2015	Male (castrated)	Jack Russell Terrier	broken	Non-carrier
JRT332	2013	Female (spayed)	Jack Russell Terrier	smooth	Non-carrier
JRT333	2004	Male (castrated)	Jack Russell Terrier	smooth	Non-carrier
JRT334	2008	Female (spayed)	Jack Russell Terrier	rough	Non-carrier
JRT335	2018	Male	Jack Russell Terrier	broken	Non-carrier
JRT336	2015	Male (castrated)	Jack Russell Terrier	rough	Non-carrier
JRT337	2019	Female (spayed)	Jack Russell Terrier	broken	Non-carrier
JRT338	2009	Male	Jack Russell Terrier	broken	Non-carrier
JRT339	2005	Male (castrated)	Jack Russell Terrier	smooth	Non-carrier
JRT340	2009	Female	Jack Russell Terrier	broken	Non-carrier
JRT341	2017	Male (castrated)	Jack Russell Terrier	broken	Non-carrier
JRT342	2009	Female (spayed)	Jack Russell Terrier	rough	Carrier
JRT343	2010	Male	Jack Russell Terrier	smooth	Non-carrier
JRT344	2015	Female (spayed)	Jack Russell Terrier	smooth	Non-carrier
JRT345	2012	Male (castrated)	Jack Russell Terrier	smooth	Non-carrier
JRT346	2014	Male (castrated)	Jack Russell Terrier	smooth	Non-carrier
JRT347	2006	Female (spayed)	Jack Russell Terrier	smooth	Non-carrier
JRT348	2006	Male (castrated)	Jack Russell Terrier	smooth	Non-carrier
JRT349	2007	Female (spayed)	Jack Russell Terrier	rough	Non-carrier
JRT350	2009	Male (castrated)	Jack Russell Terrier	N.D.	Non-carrier
JRT351	2017	Female (spayed)	Jack Russell Terrier	N.D.	Non-carrier
JRT352	2005	Female (spayed)	Jack Russell Terrier	broken	Non-carrier
JRT353	2012	Male (castrated)	Jack Russell Terrier	rough	Non-carrier
JRT354	2004	Female (spayed)	Jack Russell Terrier	smooth	Non-carrier
JRT355	2017	Female (spayed)	Jack Russell Terrier	rough	Non-carrier
JRT356	2013	Female (spayed)	Jack Russell Terrier	smooth	Non-carrier
JRT357	2007	Male	Jack Russell Terrier	smooth	Non-carrier
JRT358	2011	Male (castrated)	Jack Russell Terrier	broken	Non-carrier
JRT359	2010	Male	Jack Russell Terrier	smooth	Non-carrier
JRT360	2013	Male (castrated)	Jack Russell Terrier	broken	Non-carrier
JRT361	2007	Male (castrated)	Jack Russell Terrier	rough	Non-carrier
JRT362	2007	Female (spayed)	Jack Russell Terrier	smooth	Non-carrier
JRT363	2010	Female (spayed)	Jack Russell Terrier	smooth	Non-carrier
JRT364	2007	Female	Jack Russell Terrier	smooth	Non-carrier
JRT365	2012	Female	Jack Russell Terrier	smooth	Non-carrier
JRT366	2010	Male (castrated)	Jack Russell Terrier	smooth	Non-carrier
JRT367	2007	Female (spayed)	Jack Russell Terrier	smooth	Non-carrier
JRT368	2008	Male	Jack Russell Terrier	smooth	Non-carrier
JRT369	2007	Male	Jack Russell Terrier	rough	Non-carrier
JRT370	2010	Female	Jack Russell Terrier	rough	Non-carrier
JRT371	2015	Female	Jack Russell Terrier	rough	Non-carrier
JRT372	2008	Male (castrated)	Jack Russell Terrier	rough	Non-carrier
JRT373	2017	Male	Jack Russell Terrier	rough	Non-carrier
JRT374	2019	Male	Jack Russell Terrier	N.D.	Non-carrier
JRT375	2009	Female (spayed)	Jack Russell Terrier	smooth	Non-carrier
JRT376	2010	Male (castrated)	Jack Russell Terrier	smooth	Non-carrier
JRT377	2017	Male (castrated)	Jack Russell Terrier	rough	Non-carrier
JRT378	2016	Female (spayed)	Jack Russell Terrier	broken	Non-carrier
JRT379	2014	Male	Jack Russell Terrier	smooth	Non-carrier
JRT380	2007	Female (spayed)	Jack Russell Terrier	N.D.	Non-carrier
JRT381	2006	Female (spayed)	Jack Russell Terrier	rough	Non-carrier
JRT382	2008	Female	Jack Russell Terrier	rough	Non-carrier
JRT383	2015	Female (spayed)	Jack Russell Terrier	broken	Non-carrier
JRT384	2018	Female (spayed)	Jack Russell Terrier	smooth	Non-carrier
JRT385	2018	Male (castrated)	Jack Russell Terrier	broken	Non-carrier
JRT386	2012	Male	Jack Russell Terrier	smooth	Non-carrier
JRT387	2010	Male	Jack Russell Terrier	rough	Non-carrier
JRT388	2010	Female (spayed)	Jack Russell Terrier	broken	Non-carrier
JRT389	2009	Male (castrated)	Jack Russell Terrier	smooth	Non-carrier
JRT390	2007	Female (spayed)	Jack Russell Terrier	rough	Non-carrier
JRT391	2016	Male (castrated)	Jack Russell Terrier	rough	Non-carrier
JRT392	2013	Male (castrated)	Jack Russell Terrier	broken	Non-carrier
JRT393	2009	Female (spayed)	Jack Russell Terrier	broken	Non-carrier
JRT394	2011	Male (castrated)	Jack Russell Terrier	rough	Non-carrier
JRT395	2012	Male (castrated)	Jack Russell Terrier	smooth	Non-carrier
JRT396	2011	Male	Jack Russell Terrier	broken	Non-carrier
JRT397	2013	Female (spayed)	Jack Russell Terrier	smooth	Non-carrier
JRT398	2007	Female (spayed)	Jack Russell Terrier	smooth	Non-carrier
JRT399	2012	Female (spayed)	Jack Russell Terrier	broken	Non-carrier
JRT400	2014	Male (castrated)	Jack Russell Terrier	rough	Non-carrier
JRT401	2014	Male (castrated)	Jack Russell Terrier	smooth	Non-carrier
JRT402	2013	Male	Jack Russell Terrier	smooth	Non-carrier
JRT403	2017	Female (spayed)	Jack Russell Terrier	rough	Non-carrier
JRT404	2009	Female (spayed)	Jack Russell Terrier	smooth	Non-carrier

JRT405	2017	Female (spayed)	Jack Russell Terrier	rough	Non-carrier
JRT406	2006	Male (castrated)	Jack Russell Terrier	rough	Non-carrier
JRT407	2008	Male	Jack Russell Terrier	smooth	Non-carrier
JRT408	2008	Female	Jack Russell Terrier	smooth	Non-carrier
JRT409	2019	Female	Jack Russell Terrier	smooth	Non-carrier
JRT410	2017	Male (castrated)	Jack Russell Terrier	rough	Non-carrier
JRT411	2011	Male	Jack Russell Terrier	rough	Non-carrier
JRT412	2012	Male	Jack Russell Terrier	smooth	Non-carrier
JRT413	2014	Male (castrated)	Jack Russell Terrier	smooth	Non-carrier
JRT414	2010	Female	Jack Russell Terrier	smooth	Non-carrier
JRT415	2013	Female	Jack Russell Terrier	smooth	Non-carrier
JRT416	2004	Female	Jack Russell Terrier	smooth	Non-carrier
JRT417	2020	Female	Jack Russell Terrier	smooth	Non-carrier
JRT418	2006	Male	Jack Russell Terrier	smooth	Non-carrier
JRT419	2011	Female (spayed)	Jack Russell Terrier	rough	Non-carrier
JRT420	2012	Female (spayed)	Jack Russell Terrier	smooth	Non-carrier
JRT421	2014	Female (spayed)	Jack Russell Terrier	smooth	Non-carrier
JRT422	2014	Female (spayed)	Jack Russell Terrier	smooth	Non-carrier
JRT423	2019	Female (spayed)	Jack Russell Terrier	smooth	Non-carrier
JRT424	2018	Female (spayed)	Jack Russell Terrier	N.D.	Non-carrier
JRT425	2015	Male (castrated)	Jack Russell Terrier	rough	Non-carrier
JRT426	2017	Female (spayed)	Jack Russell Terrier	broken	Non-carrier
JRT427	2019	Male	Jack Russell Terrier	rough	Non-carrier
JRT428	2017	Male (castrated)	Jack Russell Terrier	broken	Non-carrier
JRT429	2015	Male (castrated)	Jack Russell Terrier	broken	Non-carrier
JRT430	2013	Male (castrated)	Jack Russell Terrier	broken	Non-carrier
JRT431	2006	Female	Jack Russell Terrier	smooth	Non-carrier
JRT432	2018	Male	Jack Russell Terrier	smooth	Non-carrier
JRT433	2008	Male (castrated)	Jack Russell Terrier	broken	Non-carrier
JRT434	2010	Female	Jack Russell Terrier	smooth	Non-carrier
JRT435	2007	Female (spayed)	Jack Russell Terrier	broken	Non-carrier
JRT436	2008	Female (spayed)	Jack Russell Terrier	broken	Non-carrier
JRT437	2007	Female (spayed)	Jack Russell Terrier	smooth	Non-carrier
JRT438	2016	Male (castrated)	Jack Russell Terrier	N.D.	Non-carrier
JRT439	2016	Male (castrated)	Jack Russell Terrier	N.D.	Non-carrier
JRT440	2007	Female (spayed)	Jack Russell Terrier	broken	Non-carrier
JRT441	2014	Female (spayed)	Jack Russell Terrier	smooth	Non-carrier
JRT442	2018	Male (castrated)	Jack Russell Terrier	broken	Non-carrier
JRT443	2018	Male	Jack Russell Terrier	smooth	Non-carrier
JRT444	2013	Female	Jack Russell Terrier	broken	Non-carrier
JRT445	2017	Male (castrated)	Jack Russell Terrier	rough	Non-carrier
JRT446	2015	Male	Jack Russell Terrier	smooth	Non-carrier
JRT447	2017	Male (castrated)	Jack Russell Terrier	rough	Carrier
JRT448	2007	Female (spayed)	Jack Russell Terrier	broken	Non-carrier
JRT449	2008	Female (spayed)	Jack Russell Terrier	broken	Non-carrier
JRT450	2012	Male (castrated)	Jack Russell Terrier	smooth	Non-carrier
JRT451	2011	Female (spayed)	Jack Russell Terrier	smooth	Non-carrier
JRT452	2016	Female	Jack Russell Terrier	smooth	Non-carrier
JRT453	2007	Female (spayed)	Jack Russell Terrier	smooth	Non-carrier
JRT454	2007	Male (castrated)	Jack Russell Terrier	smooth	Non-carrier
JRT455	2012	Male (castrated)	Jack Russell Terrier	broken	Non-carrier
JRT456	2019	Male	Jack Russell Terrier	rough	Non-carrier
JRT457	2014	Female (spayed)	Jack Russell Terrier	smooth	Non-carrier
JRT458	2008	Female	Jack Russell Terrier	broken	Non-carrier
JRT459	2018	Male (castrated)	Jack Russell Terrier	smooth	Non-carrier
JRT460	2009	Male	Jack Russell Terrier	N.D.	Non-carrier
JRT461	2009	Male (castrated)	Jack Russell Terrier	smooth	Non-carrier
JRT462	2003	Female (spayed)	Jack Russell Terrier	smooth	Non-carrier
JRT463	2006	Male (castrated)	Jack Russell Terrier	smooth	Non-carrier
JRT464	2007	Female (spayed)	Jack Russell Terrier	smooth	Non-carrier
JRT465	2013	Female (spayed)	Jack Russell Terrier	smooth	Non-carrier
JRT466	2014	Female (spayed)	Jack Russell Terrier	broken	Non-carrier
JRT467	2013	Male (castrated)	Jack Russell Terrier	smooth	Non-carrier
JRT468	2019	Male	Jack Russell Terrier	N.D.	Non-carrier
JRT469	2010	Female (spayed)	Jack Russell Terrier	N.D.	Non-carrier
JRT470	2017	Female (spayed)	Jack Russell Terrier	N.D.	Non-carrier
JRT471	2016	Male (castrated)	Jack Russell Terrier	N.D.	Non-carrier
JRT472	2015	Female (spayed)	Jack Russell Terrier	broken	Non-carrier
JRT473	2017	Female (spayed)	Jack Russell Terrier	rough	Non-carrier
JRT474	2019	Male	Jack Russell Terrier	smooth	Non-carrier
JRT475	2017	Female (unspecified)	Jack Russell Terrier	smooth	Non-carrier
JRT476	2004	Female (spayed)	Jack Russell Terrier	rough	Non-carrier
JRT477	2008	Female	Jack Russell Terrier	smooth	Non-carrier
JRT478	2012	Male	Jack Russell Terrier	rough	Non-carrier
JRT479	2009	Female (spayed)	Jack Russell Terrier	smooth	Non-carrier
JRT480	2010	Male (castrated)	Jack Russell Terrier	smooth	Non-carrier
JRT481	2015	Male	Jack Russell Terrier	smooth	Non-carrier
JRT482	2006	Female (spayed)	Jack Russell Terrier	broken	Non-carrier
JRT483	2019	Male	Jack Russell Terrier	broken	Non-carrier
JRT484	2018	Female (spayed)	Jack Russell Terrier	rough	Non-carrier
JRT485	2011	Male (castrated)	Jack Russell Terrier	N.D.	Non-carrier
JRT486	2012	Female (spayed)	Jack Russell Terrier	smooth	Non-carrier

JRT487	2017	Male (castrated)	Jack Russell Terrier	broken	Non-carrier
JRT488	2020	Female	Jack Russell Terrier	smooth	Non-carrier
JRT489	2005	Male	Jack Russell Terrier	smooth	Non-carrier
JRT490	2019	Female (spayed)	Jack Russell Terrier	smooth	Non-carrier
JRT491	2010	Female (spayed)	Jack Russell Terrier	broken	Non-carrier
JRT492	2007	Female (spayed)	Jack Russell Terrier	broken	Non-carrier
JRT493	2013	Male (castrated)	Jack Russell Terrier	broken	Non-carrier
JRT494	2014	Female (spayed)	Jack Russell Terrier	broken	Non-carrier
JRT495	2015	Male (castrated)	Jack Russell Terrier	broken	Non-carrier
JRT496	2005	Male (castrated)	Jack Russell Terrier	broken	Non-carrier
JRT497	2008	Female	Jack Russell Terrier	smooth	Non-carrier
JRT498	2009	Male (castrated)	Jack Russell Terrier	broken	Non-carrier
JRT499	2008	Female (spayed)	Jack Russell Terrier	broken	Non-carrier
JRT500	2004	Male (castrated)	Jack Russell Terrier	broken	Non-carrier
JRT501	2016	Female	Jack Russell Terrier	broken	Non-carrier
JRT502	2013	Female (spayed)	Jack Russell Terrier	broken	Non-carrier
JRT503	2006	Male (castrated)	Jack Russell Terrier	rough	Non-carrier
JRT504	2012	Male (castrated)	Jack Russell Terrier	smooth	Non-carrier
JRT505	2018	Male (castrated)	Jack Russell Terrier	broken	Non-carrier
JRT506	2015	Male	Jack Russell Terrier	broken	Non-carrier
JRT507	2016	Female (spayed)	Jack Russell Terrier	N.D.	Non-carrier
JRT508	2019	Male (castrated)	Jack Russell Terrier	smooth	Non-carrier
JRT509	2014	Male (castrated)	Jack Russell Terrier	broken	Non-carrier
JRT510	2014	Female (spayed)	Jack Russell Terrier	broken	Non-carrier
JRT511	2012	Male (castrated)	Jack Russell Terrier	broken	Non-carrier
JRT512	2004	Female (spayed)	Jack Russell Terrier	smooth	Non-carrier
JRT513	2017	Female (spayed)	Jack Russell Terrier	smooth	Non-carrier
JRT514	2008	Male (castrated)	Jack Russell Terrier	smooth	Non-carrier
JRT515	2012	Female (spayed)	Jack Russell Terrier	smooth	Non-carrier
JRT516	2012	Male (castrated)	Jack Russell Terrier	smooth	Non-carrier
JRT517	2018	Male (castrated)	Jack Russell Terrier	rough	Non-carrier
JRT518	2005	Male (castrated)	Jack Russell Terrier	broken	Non-carrier
JRT519	2017	Female (spayed)	Jack Russell Terrier	smooth	Non-carrier
JRT520	2011	Female	Jack Russell Terrier	smooth	Non-carrier
JRT521	2012	Female (spayed)	Jack Russell Terrier	broken	Non-carrier
JRT522	2007	Male (castrated)	Jack Russell Terrier	smooth	Non-carrier
JRT523	2012	Female (spayed)	Jack Russell Terrier	smooth	Non-carrier
JRT524	2016	Male	Jack Russell Terrier	rough	Non-carrier
JRT525	2013	Female (spayed)	Jack Russell Terrier	rough	Non-carrier
JRT526	2008	Male (castrated)	Jack Russell Terrier	rough	Non-carrier
JRT527	2006	Male (castrated)	Jack Russell Terrier	smooth	Non-carrier
JRT528	2012	Female (spayed)	Jack Russell Terrier	smooth	Non-carrier
JRT529	2008	Male (castrated)	Jack Russell Terrier	smooth	Non-carrier
JRT530	2018	Male (castrated)	Jack Russell Terrier	broken	Non-carrier
JRT531	2014	Female (spayed)	Jack Russell Terrier	smooth	Non-carrier
JRT532	2013	Female (spayed)	Jack Russell Terrier	smooth	Non-carrier
JRT533	2013	Female (spayed)	Jack Russell Terrier	rough	Non-carrier
JRT534	2019	Male (castrated)	Jack Russell Terrier	smooth	Non-carrier
JRT535	2015	Male (castrated)	Jack Russell Terrier	smooth	Carrier
JRT536	2014	Female	Jack Russell Terrier	broken	Non-carrier
JRT537	2008	Male (castrated)	Jack Russell Terrier	smooth	Non-carrier
JRT538	2007	Male (castrated)	Jack Russell Terrier	N.D.	Non-carrier
JRT539	2016	Male (castrated)	Jack Russell Terrier	smooth	Non-carrier
JRT540	2007	Male (castrated)	Jack Russell Terrier	smooth	Non-carrier
JRT541	2013	Female (spayed)	Jack Russell Terrier	broken	Non-carrier
JRT542	2018	Male (castrated)	Jack Russell Terrier	rough	Non-carrier
JRT543	2011	Female (spayed)	Jack Russell Terrier	smooth	Non-carrier
JRT544	2014	Male	Jack Russell Terrier	smooth	Non-carrier
JRT545	2019	Male (castrated)	Jack Russell Terrier	smooth	Non-carrier
JRT546	2014	Male (castrated)	Jack Russell Terrier	N.D.	Non-carrier
JRT547	2019	Female	Jack Russell Terrier	N.D.	Non-carrier
JRT548	2018	Male (castrated)	Jack Russell Terrier	rough	Non-carrier
JRT549	2019	Male	Jack Russell Terrier	rough	Non-carrier
JRT550	2017	Female (spayed)	Jack Russell Terrier	smooth	Non-carrier
JRT551	2009	Male (castrated)	Jack Russell Terrier	broken	Non-carrier
JRT552	2018	Male (castrated)	Jack Russell Terrier	rough	Non-carrier
JRT553	2016	Male	Jack Russell Terrier	smooth	Non-carrier
JRT554	2019	Female (spayed)	Jack Russell Terrier	smooth	Non-carrier
JRT555	2019	Female	Jack Russell Terrier	smooth	Non-carrier
JRT556	2005	Female (spayed)	Jack Russell Terrier	smooth	Non-carrier
JRT557	2016	Male (castrated)	Jack Russell Terrier	smooth	Non-carrier
JRT558	2008	Male	Jack Russell Terrier	broken	Non-carrier
JRT559	2011	Male	Jack Russell Terrier	broken	Non-carrier
JRT560	2017	Male	Jack Russell Terrier	smooth	Non-carrier
JRT561	2011	Female (spayed)	Jack Russell Terrier	broken	Non-carrier
JRT562	2016	Female (spayed)	Jack Russell Terrier	smooth	Non-carrier
JRT563	2010	Female (spayed)	Jack Russell Terrier	broken	Non-carrier
JRT564	2008	Male (castrated)	Jack Russell Terrier	smooth	Non-carrier
JRT565	2018	Female	Jack Russell Terrier	N.D.	Non-carrier
JRT566	2020	Female	Jack Russell Terrier	N.D.	Non-carrier
JRT567	2020	Female	Jack Russell Terrier	N.D.	Non-carrier
JRT568	2020	Male	Jack Russell Terrier	N.D.	Non-carrier

JRT569	2020	Male	Jack Russell Terrier	N.D.	Non-carrier
JRT570	2014	Female (spayed)	Jack Russell Terrier	broken	Non-carrier
JRT571	2007	Female (spayed)	Jack Russell Terrier	rough	Non-carrier
JRT572	2018	Female	Jack Russell Terrier	smooth	Non-carrier
JRT573	2014	Male (castrated)	Jack Russell Terrier	broken	Non-carrier
JRT574	2013	Female (spayed)	Jack Russell Terrier	broken	Non-carrier
JRT575	2014	Female (spayed)	Jack Russell Terrier	broken	Non-carrier
JRT576	2009	Male (castrated)	Jack Russell Terrier	smooth	Non-carrier
JRT577	2011	Male (castrated)	Jack Russell Terrier	rough	Non-carrier
JRT578	2010	Female (spayed)	Jack Russell Terrier	rough	Non-carrier
JRT579	2009	Female (spayed)	Jack Russell Terrier	smooth	Non-carrier
JRT580	2010	Female (spayed)	Jack Russell Terrier	broken	Non-carrier
JRT581	2014	Female	Jack Russell Terrier	broken	Non-carrier
JRT582	2007	Female (spayed)	Jack Russell Terrier	N.D.	Non-carrier
JRT583	2007	Female	Jack Russell Terrier	smooth	Non-carrier
JRT584	2017	Male (castrated)	Jack Russell Terrier	smooth	Non-carrier
JRT585	2004	Male (castrated)	Jack Russell Terrier	smooth	Non-carrier
JRT586	2020	Male	Jack Russell Terrier	broken	Non-carrier
JRT587	2009	Female	Jack Russell Terrier	rough	Non-carrier
JRT588	2008	Female	Jack Russell Terrier	smooth	Non-carrier
JRT589	2011	Male (castrated)	Jack Russell Terrier	broken	Non-carrier
JRT590	2011	Male (castrated)	Jack Russell Terrier	smooth	Non-carrier
JRT591	2011	Female	Jack Russell Terrier	smooth	Non-carrier
JRT592	2012	Female (spayed)	Jack Russell Terrier	smooth	Non-carrier
JRT593	2019	Male	Jack Russell Terrier	N.D.	Non-carrier
JRT594	2010	Female (spayed)	Jack Russell Terrier	smooth	Non-carrier
JRT595	2012	Female (spayed)	Jack Russell Terrier	smooth	Non-carrier
JRT596	2019	Female	Jack Russell Terrier	smooth	Non-carrier
JRT597	2018	Male (castrated)	Jack Russell Terrier	smooth	Non-carrier
JRT598	2008	Female (spayed)	Jack Russell Terrier	smooth	Non-carrier
JRT599	2007	Female (spayed)	Jack Russell Terrier	smooth	Non-carrier
JRT600	2006	Male (castrated)	Jack Russell Terrier	smooth	Non-carrier
JRT601	2014	Male (castrated)	Jack Russell Terrier	broken	Non-carrier
JRT602	2019	Female (spayed)	Jack Russell Terrier	smooth	Non-carrier
JRT603	2005	Female (spayed)	Jack Russell Terrier	smooth	Non-carrier
JRT604	2020	Male	Jack Russell Terrier	smooth	Non-carrier
JRT605	2013	Male (castrated)	Jack Russell Terrier	smooth	Non-carrier
JRT606	2014	Male (castrated)	Jack Russell Terrier	rough	Non-carrier
JRT607	2020	Female	Jack Russell Terrier	smooth	Non-carrier
JRT608	2011	Female	Jack Russell Terrier	rough	Non-carrier
JRT609	2006	Male	Jack Russell Terrier	broken	Non-carrier
JRT610	2005	Female	Jack Russell Terrier	smooth	Non-carrier
JRT611	2012	Female (spayed)	Jack Russell Terrier	broken	Non-carrier
JRT612	2007	Male (castrated)	Jack Russell Terrier	smooth	Non-carrier
JRT613	2017	Male (castrated)	Jack Russell Terrier	smooth	Non-carrier
JRT614	2018	Male (castrated)	Jack Russell Terrier	smooth	Non-carrier
JRT615	2019	Male (castrated)	Jack Russell Terrier	smooth	Non-carrier
JRT616	2012	Female (spayed)	Jack Russell Terrier	smooth	Non-carrier
JRT617	2005	Male (castrated)	Jack Russell Terrier	broken	Non-carrier
JRT618	2009	Female (spayed)	Jack Russell Terrier	broken	Non-carrier
JRT619	2015	Male (castrated)	Jack Russell Terrier	rough	Non-carrier
JRT620	2010	Female	Jack Russell Terrier	smooth	Non-carrier
JRT621	2006	Male (castrated)	Jack Russell Terrier	smooth	Non-carrier
JRT622	2004	Female (spayed)	Jack Russell Terrier	smooth	Non-carrier
JRT623	2008	Female (spayed)	Jack Russell Terrier	broken	Non-carrier
JRT624	2011	Male (castrated)	Jack Russell Terrier	rough	Non-carrier
JRT625	2012	Female (spayed)	Jack Russell Terrier	smooth	Non-carrier
JRT626	2015	Male (castrated)	Jack Russell Terrier	broken	Non-carrier
JRT627	2012	Male (castrated)	Jack Russell Terrier	smooth	Non-carrier
JRT628	2009	Female (spayed)	Jack Russell Terrier	broken	Non-carrier
JRT629	2016	Female (spayed)	Jack Russell Terrier	smooth	Non-carrier
JRT630	2013	Male (castrated)	Jack Russell Terrier	smooth	Non-carrier
JRT631	2012	Male	Jack Russell Terrier	smooth	Non-carrier
JRT632	2007	Female (spayed)	Jack Russell Terrier	smooth	Non-carrier
JRT633	2004	Male (castrated)	Jack Russell Terrier	smooth	Non-carrier
JRT634	2009	Female	Jack Russell Terrier	rough	Non-carrier
JRT635	2019	Male	Jack Russell Terrier	smooth	Non-carrier
JRT636	2009	Male	Jack Russell Terrier	smooth	Non-carrier
JRT637	2011	Female (spayed)	Jack Russell Terrier	broken	Non-carrier
JRT638	2016	Male (castrated)	Jack Russell Terrier	broken	Non-carrier
JRT639	2014	Female (spayed)	Jack Russell Terrier	smooth	Non-carrier
JRT640	2010	Female (spayed)	Jack Russell Terrier	smooth	Non-carrier
JRT641	2015	Male	Jack Russell Terrier	smooth	Non-carrier
JRT642	2015	Female (spayed)	Jack Russell Terrier	smooth	Non-carrier
JRT643	2005	Male	Jack Russell Terrier	rough	Non-carrier
JRT644	2009	Female (spayed)	Jack Russell Terrier	smooth	Non-carrier
JRT645	2011	Female (spayed)	Jack Russell Terrier	broken	Non-carrier
JRT646	2010	Male	Jack Russell Terrier	N.D.	Non-carrier
JRT647	2007	Female (spayed)	Jack Russell Terrier	smooth	Non-carrier
JRT648	2013	Female (spayed)	Jack Russell Terrier	smooth	Non-carrier
JRT649	2012	Female (spayed)	Jack Russell Terrier	smooth	Non-carrier
JRT650	2006	Female	Jack Russell Terrier	smooth	Non-carrier

JRT651	2013	Male (castrated)	Jack Russell Terrier	smooth	Non-carrier
JRT652	2012	Male	Jack Russell Terrier	smooth	Non-carrier
JRT653	2008	Male	Jack Russell Terrier	smooth	Non-carrier
JRT654	2010	Female	Jack Russell Terrier	smooth	Non-carrier
JRT655	2009	Male	Jack Russell Terrier	smooth	Non-carrier
JRT656	2015	Female (spayed)	Jack Russell Terrier	smooth	Non-carrier
JRT657	2011	Male (castrated)	Jack Russell Terrier	broken	Non-carrier
JRT658	2010	Male	Jack Russell Terrier	smooth	Non-carrier
JRT659	2019	Male (castrated)	Jack Russell Terrier	broken	Non-carrier
JRT660	2006	Male (castrated)	Jack Russell Terrier	smooth	Non-carrier
JRT661	2011	Female (spayed)	Jack Russell Terrier	broken	Non-carrier
JRT662	2017	Male (castrated)	Jack Russell Terrier	smooth	Non-carrier
JRT663	2014	Male(unspecified)	Jack Russell Terrier	smooth	Non-carrier
JRT664	2015	Female (spayed)	Jack Russell Terrier	rough	Non-carrier
JRT665	2019	Male(unspecified)	Jack Russell Terrier	smooth	Non-carrier
JRT666	2008	Male (castrated)	Jack Russell Terrier	broken	Non-carrier
JRT667	2010	Female (spayed)	Jack Russell Terrier	smooth	Non-carrier
JRT668	2011	Female	Jack Russell Terrier	smooth	Non-carrier
JRT669	2009	Male	Jack Russell Terrier	smooth	Non-carrier
JRT670	2017	Male	Jack Russell Terrier	smooth	Non-carrier
JRT671	2011	Male (castrated)	Jack Russell Terrier	smooth	Carrier
JRT672	2015	Male (castrated)	Jack Russell Terrier	smooth	Non-carrier
JRT673	2017	Male (castrated)	Jack Russell Terrier	broken	Non-carrier
JRT674	2009	Male	Jack Russell Terrier	smooth	Non-carrier
JRT675	2014	Female (spayed)	Jack Russell Terrier	smooth	Non-carrier
JRT676	2017	Male (castrated)	Jack Russell Terrier	broken	Non-carrier
JRT677	2017	Female (spayed)	Jack Russell Terrier	smooth	Non-carrier
JRT678	2019	Male (castrated)	Jack Russell Terrier	rough	Non-carrier
JRT679	2017	Female (spayed)	Jack Russell Terrier	broken	Non-carrier
JRT680	2012	Male (castrated)	Jack Russell Terrier	smooth	Non-carrier
JRT681	2013	Female (spayed)	Jack Russell Terrier	broken	Non-carrier
JRT682	2007	Female (spayed)	Jack Russell Terrier	rough	Non-carrier
JRT683	2015	Male (castrated)	Jack Russell Terrier	broken	Non-carrier
JRT684	2016	Male (castrated)	Jack Russell Terrier	smooth	Non-carrier
JRT685	2005	Male (castrated)	Jack Russell Terrier	smooth	Non-carrier
JRT686	2006	Male (castrated)	Jack Russell Terrier	smooth	Non-carrier
JRT687	2018	Male (castrated)	Jack Russell Terrier	smooth	Non-carrier
JRT688	2011	Female	Jack Russell Terrier	smooth	Non-carrier
JRT689	2007	Female (spayed)	Jack Russell Terrier	smooth	Non-carrier
JRT690	2015	Female (spayed)	Jack Russell Terrier	smooth	Non-carrier
JRT691	2013	Female (spayed)	Jack Russell Terrier	rough	Non-carrier
JRT692	2009	Male	Jack Russell Terrier	smooth	Non-carrier
JRT693	2010	Male	Jack Russell Terrier	smooth	Non-carrier
JRT694	2016	Female (spayed)	Jack Russell Terrier	smooth	Non-carrier
JRT695	2019	Female (spayed)	Jack Russell Terrier	smooth	Non-carrier
JRT696	2011	Female (spayed)	Jack Russell Terrier	broken	Non-carrier
JRT697	2007	Female (spayed)	Jack Russell Terrier	broken	Carrier
JRT698	2005	Female (spayed)	Jack Russell Terrier	smooth	Non-carrier
JRT699	2012	Male (castrated)	Jack Russell Terrier	smooth	Non-carrier
JRT700	2019	Male (castrated)	Jack Russell Terrier	smooth	Non-carrier
JRT701	2016	Male (castrated)	Jack Russell Terrier	smooth	Non-carrier
JRT702	2007	Male (castrated)	Jack Russell Terrier	rough	Non-carrier
JRT703	2007	Male (castrated)	Jack Russell Terrier	broken	Non-carrier
JRT704	2005	Male (castrated)	Jack Russell Terrier	broken	Non-carrier
JRT705	2007	Female (spayed)	Jack Russell Terrier	broken	Non-carrier
JRT706	2007	Female (spayed)	Jack Russell Terrier	broken	Non-carrier
JRT707	2008	Male (castrated)	Jack Russell Terrier	smooth	Non-carrier
JRT708	2006	Male (castrated)	Jack Russell Terrier	broken	Non-carrier
JRT709	2014	Male (castrated)	Jack Russell Terrier	smooth	Non-carrier
JRT710	2010	Male (castrated)	Jack Russell Terrier	broken	Non-carrier
JRT711	2018	Female	Jack Russell Terrier	broken	Non-carrier
JRT712	2009	Male	Jack Russell Terrier	broken	Non-carrier
JRT713	2008	Female (spayed)	Jack Russell Terrier	broken	Non-carrier
JRT714	2015	Female	Jack Russell Terrier	broken	Non-carrier
JRT715	2004	Female (spayed)	Jack Russell Terrier	smooth	Non-carrier
JRT716	2009	Female (spayed)	Jack Russell Terrier	broken	Non-carrier
JRT717	2012	Male (castrated)	Jack Russell Terrier	broken	Non-carrier
JRT718	2013	Male (castrated)	Jack Russell Terrier	N.D.	Non-carrier
JRT719	2014	Female (spayed)	Jack Russell Terrier	rough	Non-carrier
JRT720	2011	Female (spayed)	Jack Russell Terrier	rough	Non-carrier
JRT721	2009	Male (castrated)	Jack Russell Terrier	smooth	Non-carrier
JRT722	2010	Female (spayed)	Jack Russell Terrier	rough	Non-carrier
JRT723	2014	Male	Jack Russell Terrier	smooth	Non-carrier
JRT724	2006	Female (spayed)	Jack Russell Terrier	rough	Non-carrier
JRT725	2017	Male (castrated)	Jack Russell Terrier	smooth	Non-carrier
JRT726	2010	Male (castrated)	Jack Russell Terrier	rough	Non-carrier
JRT727	2015	Female (spayed)	Jack Russell Terrier	smooth	Non-carrier
JRT728	2009	Male	Jack Russell Terrier	smooth	Non-carrier
JRT729	2014	Male	Jack Russell Terrier	smooth	Non-carrier
JRT730	2014	Male (castrated)	Jack Russell Terrier	rough	Non-carrier
JRT731	2011	Male (castrated)	Jack Russell Terrier	rough	Non-carrier
JRT732	2017	Male (castrated)	Jack Russell Terrier	broken	Non-carrier

JRT733	2007	Female (spayed)	Jack Russell Terrier	broken	Non-carrier
JRT734	2007	Female (spayed)	Jack Russell Terrier	smooth	Non-carrier
JRT735	2019	Male (castrated)	Jack Russell Terrier	smooth	Non-carrier
JRT736	2008	Male (castrated)	Jack Russell Terrier	N.D.	Non-carrier
JRT737	2013	Male (castrated)	Jack Russell Terrier	smooth	Non-carrier
JRT738	2007	Male (castrated)	Jack Russell Terrier	smooth	Non-carrier
JRT739	2008	Female (spayed)	Jack Russell Terrier	smooth	Non-carrier
JRT740	2010	Female (spayed)	Jack Russell Terrier	smooth	Non-carrier
JRT741	2012	Male	Jack Russell Terrier	N.D.	Non-carrier
JRT742	2009	Male	Jack Russell Terrier	broken	Non-carrier
JRT743	2015	Male	Jack Russell Terrier	N.D.	Non-carrier
JRT744	2008	Female (spayed)	Jack Russell Terrier	smooth	Non-carrier
JRT745	2014	Female (spayed)	Jack Russell Terrier	rough	Non-carrier
JRT746	2016	Female (spayed)	Jack Russell Terrier	broken	Non-carrier
JRT747	2007	Female	Jack Russell Terrier	broken	Non-carrier
JRT748	2012	Male (castrated)	Jack Russell Terrier	smooth	Non-carrier
JRT749	2017	Male	Jack Russell Terrier	rough	Non-carrier
JRT750	2015	Female (spayed)	Jack Russell Terrier	broken	Non-carrier
JRT751	2011	Male	Jack Russell Terrier	broken	Non-carrier
JRT752	2012	Female	Jack Russell Terrier	smooth	Non-carrier
JRT753	2013	Female (spayed)	Jack Russell Terrier	broken	Non-carrier
JRT754	2007	Female (spayed)	Jack Russell Terrier	smooth	Non-carrier
JRT755	2006	Female (spayed)	Jack Russell Terrier	smooth	Non-carrier
JRT756	2010	Female (spayed)	Jack Russell Terrier	smooth	Non-carrier
JRT757	2009	Male	Jack Russell Terrier	rough	Non-carrier
JRT758	2014	Male	Jack Russell Terrier	smooth	Non-carrier
JRT759	2006	Female	Jack Russell Terrier	broken	Non-carrier
JRT760	2017	Female (spayed)	Jack Russell Terrier	smooth	Non-carrier
JRT761	2010	Female (spayed)	Jack Russell Terrier	smooth	Non-carrier
JRT762	2011	Male	Jack Russell Terrier	smooth	Non-carrier
JRT763	2011	Female (spayed)	Jack Russell Terrier	smooth	Non-carrier
JRT764	2011	Male (castrated)	Jack Russell Terrier	broken	Non-carrier
JRT765	2009	Female (spayed)	Jack Russell Terrier	smooth	Non-carrier
JRT766	2007	Female (spayed)	Jack Russell Terrier	broken	Non-carrier
JRT767	2016	Female (spayed)	Jack Russell Terrier	broken	Non-carrier
JRT768	2018	Male (castrated)	Jack Russell Terrier	N.D.	Non-carrier
JRT769	2017	Female (spayed)	Jack Russell Terrier	smooth	Non-carrier
JRT770	2009	Male	Jack Russell Terrier	smooth	Non-carrier
JRT771	2011	Male	Jack Russell Terrier	smooth	Non-carrier
JRT772	2011	Female (spayed)	Jack Russell Terrier	smooth	Non-carrier
JRT773	2004	Male (castrated)	Jack Russell Terrier	smooth	Non-carrier
JRT774	2006	Female (spayed)	Jack Russell Terrier	smooth	Non-carrier
JRT775	2008	Male	Jack Russell Terrier	smooth	Non-carrier
JRT776	2019	Female (spayed)	Jack Russell Terrier	smooth	Non-carrier
JRT777	2009	Female	Jack Russell Terrier	smooth	Non-carrier
JRT778	2010	Male	Jack Russell Terrier	smooth	Non-carrier
JRT779	2007	Male (castrated)	Jack Russell Terrier	broken	Non-carrier
JRT780	2019	Male	Jack Russell Terrier	smooth	Non-carrier
JRT781	2009	Female (spayed)	Jack Russell Terrier	smooth	Non-carrier
JRT782	2009	Female (spayed)	Jack Russell Terrier	smooth	Non-carrier
JRT783	2014	Male	Jack Russell Terrier	rough	Non-carrier
JRT784	2018	Male	Jack Russell Terrier	smooth	Non-carrier
JRT785	2008	Female (spayed)	Jack Russell Terrier	broken	Non-carrier
JRT786	2009	Male (castrated)	Jack Russell Terrier	broken	Non-carrier
JRT787	2015	Female	Jack Russell Terrier	rough	Non-carrier
JRT788	2013	Female (spayed)	Jack Russell Terrier	rough	Non-carrier
JRT789	2007	Female (spayed)	Jack Russell Terrier	broken	Non-carrier
JRT790	N.D.	Female (spayed)	Jack Russell Terrier	broken	Non-carrier
JRT791	N.D.	Male (castrated)	Jack Russell Terrier	rough	Non-carrier
JRT792	2018	Female (spayed)	Jack Russell Terrier	rough	Non-carrier

Previous cases of Jack Russell Terriers with hereditary gastrointestinal polyposis used in pedigree analysis

Case No.	Birth year	Sex	Dog breed	Location and histopatological diagnosis of gastrointestinal polyps	*APC* variant status	Reference
JRT-P01	2005/09	Male (castrated)	Jack Russell Terrier	Large intestine: adenocarcinoma (n=1)	Carrier	[6]
JRT-P02	2005/12	Female (spayed)	Jack Russell Terrier	Large intestine: adenocarcinoma (n=1)	Carrier	[6]
JRT-P03	2006/05	Female (spayed)	Jack Russell Terrier	Stomach: adenoma (n=1), adenocarcinoma (n=2); large intestine: adenocarcinoma (n=2)	Carrier	[6]
JRT-P04	2010/04	Female	Jack Russell Terrier	Stomach: adenocarcinoma (n=1)	Carrier	[6]
JRT-P05	2010/07	Female (spayed)	Jack Russell Terrier	Stomach: adenocarcinoma (n=2)	Carrier	[6]

Characteristics, pathological information, and APC variant status of dogs with gastrointestinal epithelial tumor of multiple breeds in retrospective analysis

Case No.	Age at diagnosis	Sex	Dog breed	Location of GI epithelial tumor	Histopathological diagnosis	*APC* variant status
FFPE001	9 y 2 m	Female (spayed)	Mixed-breed (Japanese dog)	Small intestine (unspecified)	Adenocarcinoma (Signet-ring cell carcinoma)	Non-carrier
FFPE002	8 y 6 m	Female (spayed)	Miniature Dachshund	Large intestine (Rectum)	Adenoma	Non-carrier
FFPE003	3 y 3 m	Female	Bull Terrier	Large intestine (Rectum)	Adenoma	Non-carrier
FFPE004	10 y 3 m	Male (castrated)	Mixed-breed	Large intestine (Rectum)	Adenoma	Non-carrier
FFPE005	8 y 8 m	Female	Toy Poodle	Large intestine (Cecum)	Adenoma	Non-carrier
FFPE006	2 y 9 m	Male	French Bulldog	Large intestine (Rectum)	Adenoma	Non-carrier
FFPE007	12 y 3 m	Male	Miniature Dachshund	Small intestine (Ileum)	Adenocarcinoma	Non-carrier
FFPE008	14 y 0 m	Male	Miniature Dachshund	Small intestine (unspecified)	Adenocarcinoma	Non-carrier
FFPE009	9 y 5 m	Male (castrated)	Welsh Corgi	Large intestine (Colon)	Adenoma	Non-carrier

FFPE010	13 y 8 m	Female (spayed)	Toy Poodle	Small intestine (unspecified)	Adenocarcinoma	Non-carrier
FFPE011	11 y	Female	Chihuahua	Large intestine (Cecum)	Adenoma	Non-carrier
FFPE012	10 y	Male	Chihuahua	Large intestine (unspecified)	Adenocarcinoma	Non-carrier
FFPE013	12 y	Male (unspecified)	French Bulldog	Stomach	Adenocarcinoma	Non-carrier
FFPE014	12 y	Male (castrated)	Shih Tzu	Small intestine (Jejunum)	Adenocarcinoma	Non-carrier
FFPE015	9 y	Male	French Bulldog	Large intestine (Rectum)	Adenocarcinoma (Signet-ring cell carcinoma)	Non-carrier
FFPE016	7 y	Female (spayed)	Maltese	Large intestine (Colon)	Adenocarcinoma	Non-carrier
FFPE017	11 y	Female	Mixed-breed (Japanese dog)	Small intestine (Ileum)	Adenocarcinoma	Non-carrier
FFPE018	12 y	Male	Mixed-breed	Large intestine (Rectum)	Adenoma	Non-carrier
FFPE019	8 y 7 m	Male	West Highland white terrier	Large intestine (Rectum)	Adenocarcinoma	Non-carrier
FFPE020	11 y 8 m	Female (spayed)	Chihuahua	Small intestine (unspecified)	Adenocarcinoma	Non-carrier
FFPE021	6 y 9 m	Male (castrated)	Toy Poodle	Large intestine (Rectum)	Adenoma	Non-carrier
FFPE022	10 y 11 m	Male (castrated)	Labrador Retriever	Large intestine (Rectum)	Adenoma	Non-carrier
FFPE023	13 y 5 m	Male (unspecified)	Shih Tzu	Small intestine (unspecified)	Adenocarcinoma	Non-carrier
FFPE024	N.D.	Female	Miniature Schnauzer	Small intestine (unspecified)	Adenocarcinoma	Non-carrier
FFPE025	7 y	Female (spayed)	Border Collie	Large intestine (unspecified)	Adenocarcinoma	Non-carrier
FFPE026	11 y	Male	Miniature Dachshund	Small intestine (Jejunum)	Adenocarcinoma	Non-carrier
FFPE027	7 y 8 m	Male (castrated)	Chihuahua	Small intestine (not specified)	Adenocarcinoma	Non-carrier
FFPE028	9 y 2 m	Male	Bichon Frise	Large intestine (Rectum)	Adenoma	Non-carrier
FFPE029	13 y 4 m	Male	Labrador Retriever	Large intestine (Rectum)	Adenoma	Non-carrier
FFPE030	14 y 2 m	N.D.	Toy Poodle	Small intestine (unspecified)	Adenocarcinoma	Non-carrier
FFPE031	9 y	Male	Toy Poodle	Small intestine (unspecified)	Adenocarcinoma	Non-carrier
FFPE032	6 y 5 m	Male	Wire Fox Terrier	Large intestine (Rectum)	Adenoma	Non-carrier

Characteristics, pathological information, and APC variant status of dogs with gastrointestinal epithelial tumors obtained from genomic bank at Azabu University

Case No.	Age at diagnosis	Sex	Dog breed	Location of GI epithelial tumor	Histopathological diagnosis	*APC* variant status
GB001	N.D.	N.D.	Miniature Dachshund	Intestine (unspecified)	Adenoma	Non-carrier
GB002	N.D.	N.D.	Miniature Dachshund	Intestine (rectum)	Adenocarcinoma	Non-carrier
GB003	N.D.	N.D.	Miniature Dachshund	Stomach	Adenocarcinoma	Non-carrier
GB004	N.D.	N.D.	Miniature Dachshund	Small intestine	Adenoma	Non-carrier
GB005	N.D.	N.D.	Belgian Shepherd Tarvuren	Stomach	Adenocarcinoma	Non-carrier
GB006	N.D.	N.D.	Papillon	Intestine (unspecified)	Adenocarcinoma	Non-carrier
GB007	N.D.	N.D.	Shetland Sheepdog	Intestine (unspecified)	Adenoma	Non-carrier
GB008	N.D.	N.D.	Jack Russell Terrier	Intestine (rectum)	Adenocarcinoma	Non-carrier
GB009	N.D.	N.D.	Miniature Dachshund	Intestine (unspecified)	Adenocarcinoma	Non-carrier
GB010	N.D.	N.D.	Miniature Dachshund	Intestine (unspecified)	Adenocarcinoma	Non-carrier
GB011	N.D.	N.D.	Toy Poodle	Intestine (rectum)	Adenocarcinoma	Non-carrier
GB012	N.D.	N.D.	Chihuahua	Intestine (unspecified)	Adenocarcinoma	Non-carrier
GB013	N.D.	N.D.	Shiba Inu	Intestine (unspecified)	Adenocarcinoma	Non-carrier
GB014	N.D.	N.D.	Boston Terrier	Intestine (rectum)	Adenoma	Non-carrier
GB015	N.D.	N.D.	Mixed-breed	Intestine (unspecified)	Adenocarcinoma	Non-carrier
GB016	N.D.	N.D.	Shih Tzu	Stomach	Adenocarcinoma	Non-carrier
GB017	N.D.	N.D.	American Cocker Spaniel	Intestine (unspecified)	Adenocarcinoma	Non-carrier
GB018	N.D.	N.D.	Jack Russell Terrier	Intestine (unspecified)	Adenocarcinoma	Carrier
GB019	N.D.	N.D.	Miniature Dachshund	Intestine (unspecified)	Adenocarcinoma	Non-carrier
GB020	N.D.	N.D.	Chinese Crested Dog	Intestine (unspecified)	Adenocarcinoma	Non-carrier
GB021	N.D.	N.D.	Miniature Dachshund	Stomach	Adenocarcinoma	Non-carrier
GB022	N.D.	N.D.	Miniature Dachshund	Stomach	Adenocarcinoma	Non-carrier
GB023	N.D.	N.D.	Chihuahua	Small intestine	Adenocarcinoma	Non-carrier
GB024	N.D.	N.D.	Lakeland Terrier	Intestine (unspecified)	Adenocarcinoma	Non-carrier
GB025	N.D.	N.D.	Miniature Schnauzer	Intestine (unspecified)	Adenocarcinoma	Non-carrier
GB026	N.D.	N.D.	Miniature Dachshund	Intestine (rectum)	Adenoma	Non-carrier
GB027	N.D.	N.D.	Miniature Dachshund	Intestine (unspecified)	Adenocarcinoma	Non-carrier
GB028	N.D.	N.D.	Golden Retriever	Stomach	Adenocarcinoma	Non-carrier
GB029	N.D.	N.D.	Toy Poodle	Intestine (unspecified)	Adenocarcinoma	Non-carrier
GB030	N.D.	N.D.	Toy Poodle	Intestine (unspecified)	Adenocarcinoma	Non-carrier
GB031	N.D.	N.D.	Miniature Dachshund	Intestine (unspecified)	Adenoma	Non-carrier
GB032	N.D.	N.D.	Shih Tzu	Stomach	Adenocarcinoma	Non-carrier
GB033	N.D.	N.D.	Jack Russell Terrier	Stomach	Adenocarcinoma	Carrier
GB034	N.D.	N.D.	Brittany Spaniel	Intestine (unspecified)	Adenocarcinoma	Non-carrier
GB035	N.D.	N.D.	Shih Tzu	Intestine (unspecified)	Adenocarcinoma	Non-carrier
GB036	N.D.	N.D.	Toy Poodle	Intestine (unspecified)	Adenocarcinoma	Non-carrier
GB037	N.D.	N.D.	French Bulldog	Stomach	Adenocarcinoma	Non-carrier
GB038	N.D.	N.D.	West Highland white terrier	Intestine (unspecified)	Adenocarcinoma	Non-carrier
GB039	N.D.	N.D.	Jack Russell Terrier	Stomach	Adenocarcinoma	Carrier

N.D.: Not data, y: years, m: months
